# The Nature of Strong Chalcogen Bonds Involving Chalcogen‐Containing Heterocycles

**DOI:** 10.1002/anie.202010309

**Published:** 2020-09-07

**Authors:** Gebhard Haberhauer, Rolf Gleiter

**Affiliations:** ^1^ Institut für Organische Chemie Universität Duisburg-Essen Universitätsstr. 7 45117 Essen Germany; ^2^ Organisch-Chemisches Institut Universität Heidelberg Im Neuenheimer Feld 270 69120 Heidelberg Germany

**Keywords:** chalcogen bonding, DFT calculations, SAPT, steric interactions

## Abstract

Chalcogen bonds are σ hole interactions and have been used in recent years as an alternative to hydrogen bonds. In general, the electrostatic potential at the chalcogen atom and orbital delocalization effects are made responsible for the orientation of the chalcogen bond. Here, we were able to show by means of SAPT calculations that neither the induction (orbital delocalization effects) nor the electrostatic term is causing the spatial orientation of strong chalcogen bonds in tellurium‐containing aromatics. Instead, steric interactions (Pauli repulsion) are responsible for the orientation. Against chemical intuition the dispersion energies of the examined tellurium‐containing aromatics are far less important for the net attractive forces compared to the energies in the corresponding sulfur and selenium compounds. Our results underline the importance of often overlooked steric interactions (Pauli repulsion) in conformational control of σ hole interactions.

## Graphical abstract

SAPT calculations of chalcogen bonds show that neither the induction (orbital delocalization effects) nor the electrostatic term is causing the spatial orientation of strong chalcogen bonds in tellurium‐containing aromatics. Instead, steric interactions (Pauli repulsion) are responsible for the orientation. These results underline the importance of often overlooked steric interactions in conformational control of σ hole interactions.

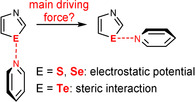

## Introduction

In recent years research in the area of chalcogen bonding^[1]^ has been absolutely boomed. Chalcogen bonds are caused by net attractive interactions between an electron‐deficient chalcogen atom E (E=O, S, Se, Te) and a Lewis base.^[1]^ In the beginning the basic concept of chalcogen bonding was focused,[^2^, ^3^, ^4^, ^5^, ^6^, ^7^, ^8^] whereas in recent times, the use of strong chalcogen bonds has become an object of interest.[^9^, ^10^, ^11^] It could be shown that this bond type can also be used for crystal engineering,[^12^, ^13^, ^14^] molecular recognition in solution[^15^, ^16^, ^17^, ^18^, ^19^, ^20^, ^21^] and catalysis.[^11^, ^20^, ^22^, ^23^, ^24^, ^25^, ^26^, ^27^, ^28^] Very strong and promising chalcogen bonds are found in electron‐poor tellurium compounds such as isotellurazole oxides[^21^, ^29^] or telluradiazole.[^18^, ^30^, ^31^] These tellurium‐containing systems form strongly associating dimers and oligomers^[21]^ in solution and can therefore be used as recognition units in supramolecular chemistry.^[18]^ In addition to isotellurazole oxides and telluradiazoles, tellurazoles have recently been used as promising building blocks for the design of strong chalcogen bonds.[^12^, ^32^] Furthermore, it could be shown that the intermolecular interactions between the tellurium atoms of tellurazoles and the oxygen atom of an amide group result in the formation of supramolecular wires and organic framework in solid state.^[33]^

Due to fundamental theoretical investigations the principle of chalcogen bonding has been understood in terms of strength and orientation.[^14^, ^34^, ^35^] Three essential components contribute to the strength of chalcogen bonds: electrostatics, orbital mixing (induction interactions) and dispersion.^[9]^ The latter is the most important term in Me_2_EEMe_2_ systems and amounts to 70–90 % of the sum of all attractive terms.[^6^, ^8^] However, experimental investigations on the conformation of chalcogen‐containing aromatics allow the conclusion that electrostatic and van der Waals dispersion forces are not responsible for the observed experimental trends.^[36]^ Instead, chalcogen bonding interactions are dominated by n→σ* orbital delocalization.^[36]^

The relative orientations of the atoms involved in a chalcogen bond are often explained by orbital mixing and/or electrostatic potential.^[9]^ Typically, the electrostatic contribution is described by a σ hole,[^35^, ^37^, ^38^] which is a region of positive electrostatic potential located on the chalcogen atom at the opposite side of the E‐R bond. The more electrons are withdrawn by the substituent R at the chalcogen atom, the more positive is the potential at the σ hole. The relative orientations of the chalcogen atom and the Lewis base having the lowest energy are such that the region with positive electrostatic potential on the outer surface of the chalcogen atom is approaching the negative region of the Lewis base. The orbital mixing contribution is designated as attractive interaction between the occupied orbital of the Lewis base (n) with the empty E‐R σ* orbital of the chalcogen atom (n(Lewis Base) → σ*(E‐R) donation). This stabilizing interaction results in a highly directed orientation of the centers involved in chalcogen bonding. Here again, electron withdrawing substituents R lower the energy level of the LUMO (lowest unoccupied molecular orbital) containing the σ* orbital, thus, making this orbital a better electron acceptor.

Considering the three attractive interactions it becomes evident that tellurium‐containing compounds can form strong chalcogen bonds:^[9]^ due to the high polarization of the tellurium atom, tellurium compounds show high dispersion interactions, deep σ holes and low‐lying LUMO orbitals.

Herein, we investigate by means of SAPT (symmetry‐adapted intermolecular perturbation theory) calculations the nature of strong chalcogen bonds, which are formed by tellurium‐containing aromatics and different Lewis bases. The main focus is on the question of which energy terms (electrostatic, induction or dispersion interaction) mostly contribute to the formation of strong chalcogen bonds. Furthermore, a model is used to clarify which interactions are responsible for spatial orientation of chalcogen bonds.

## Results and Discussion

### Analysis of Chalcogen Bonds using SAPT Calculations

**Figure 1:**
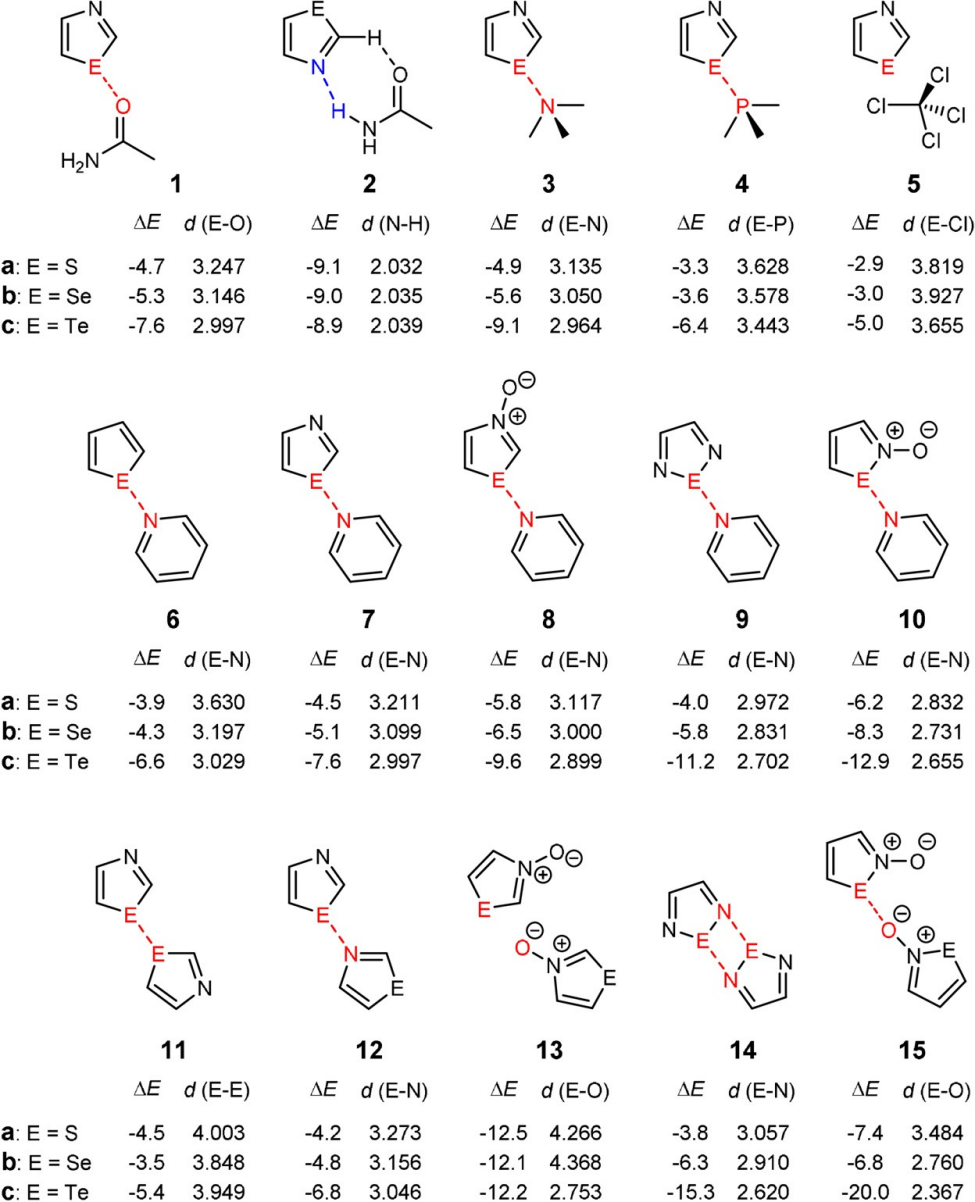
Structures of the investigated complexes between chalcogen‐containing aromatics and different Lewis bases. Data of the complex formation energies (Δ*E*) are given in kcal mol^−1^ and result from CCSD(T)/TZVP,aug‐cc‐pVTZ calculations. The distances [Å] between the chalcogen centers and the electron donor atoms are computed by means of B2PLYP‐D3/TZVP,aug‐cc‐pVTZ.

Our studies were focused on investigations of the nature of strong chalcogen bonds that exist primarily between tellurium‐containing nitrogen heterocycles and different Lewis bases.[^12^, ^15^, ^17^, ^18^, ^21^, ^30^, ^31^, ^32^, ^33^] The latter are for instance oxygen or nitrogen atoms of heterocycles or amides. In this study, we chose tellurium‐containing aromatics as model compounds and calculated the dimers and their complexes with acetamide, trimethylamine, trimethylphosphine, tetrachloromethane and pyridine (Figure 1). For comparison purposes the corresponding sulfur‐ and selenium‐containing complexes were also investigated.

In order to optimize the structures of the monomers and complexes **1**–**15** the double‐hybrid density functional B2PLYP^[39]^ was employed. In this approximation a part of the correlation energy is calculated with second‐order perturbation theory.^[39]^ As it is essential for this type of complexes to consider the dispersion interaction in an appropriate way,[^6^, ^14^, ^40^] the additional dispersion correction with Becke–Johnson damping^[41]^ (D3BJ) was used. As basis set TZVP (triple zeta valence basis set with polarization functions) was employed for the light elements C, H, N, P, O, S, Se and Cl, whereas aug‐cc‐pVTZ‐PP (augmented correlation‐consistent polarized valence triple zeta basis set) was used for tellurium. Subsequent frequency analyses show that all structures are minima on the potential energy surface. Furthermore, single point calculations on the B2PLYP‐optimized structures were performed using B3LYP,[^42^, ^43^, ^44^] B3LYP‐D3,^[41]^ B2PLYP and CCSD(T).^[45]^ As B3LYP and B3LYP‐D3 rely on the same density functional, the difference between the two is a hint to the extent of the intermolecular dispersion energies.[^46^, ^47^] The calculated data for the structures of the complexes **1**–**15** are summarized in Tables S1–S2 and Figure 1.

After a look at the geometries of the complexes it can be stated that the formation of chalcogen bonds is not happening in all cases: in case of **2 a**–**c** and **13 a**,**b** both units are connected via two hydrogen bonds (Figures S4–6 and S37–38), whereas van der Waals complexes are present between two aromatic systems (in **11 a** and **15 a**) or between an aromatic system and tetrachloromethane (in **5 a**–**c**) (Figures S13–15, S31 and S43). The CCSD(T)‐calculated formation energies for the complexes with chalcogen bonds amount to values between −3.3 and −6.2 kcal mol^−1^ in case of sulfur‐containing compounds (Figure 1). For selenium‐containing systems the formation energies of chalcogen bonds are normally 1–2 kcal mol^−1^ higher. Regarding the formation energies of tellurium‐containing aromatics there is—as expected—a remarkable leap with values up to −20 kcal mol^−1^ (**15 c**). For simplification in the following discussion the complexes are divided into two groups: those with weak chalcogen bonds (E=S, Se) and those with strong chalcogen bonds (E=Te). The energies of the complexes calculated with B2PLYP, B3LYP‐D3 and B3LYP are in fact different with regard to the magnitude of the values, but the trend due to which tellurium compounds form the strongest chalcogen bonds is still present (Tables S1–2).

In the next step we used the DFT‐SAPT[^48^, ^49^, ^50^, ^51^] (density‐functional theory – symmetry‐adapted intermolecular perturbation theory) program to calculate the total interaction energy between the units in the complexes **1**–**15**. The SAPT method yields a partitioning of the interaction energy in terms of the notions of electrostatic ($E_{ELST}^{\left(1\right)}$ ), induction ($E_{IND}^{\left(2\right)}=E_{ind}^{\left(2\right)}+E_{exch-ind}^{\left(2\right)}$ ), dispersion ($E_{DISP}^{\left(2\right)}=E_{disp}^{\left(2\right)}+E_{exch-disp}^{\left(2\right)}$ ) and exchange ($E_{EXCH}^{\left(1\right)}$ ) interactions.^[48]^ The latter term ($E_{EXCH}^{\left(1\right)}$ ) is related to the Pauli repulsion which essentially describes the repulsive component of the interaction due to the overlap of the densities of the interacting units. The Pauli repulsion has been associated with steric interaction.^[52]^ In order to include higher order contributions, we added the δ(HF) term to the SAPT interaction energy:^[48]^

$E^{SAPT}=E_{ELST}^{\left(1\right)}+E_{EXCH}^{\left(1\right)}+E_{IND}^{\left(2\right)}+E_{DISP}^{\left(2\right)}+\delta \left(HF\right)$

**Figure 2:**
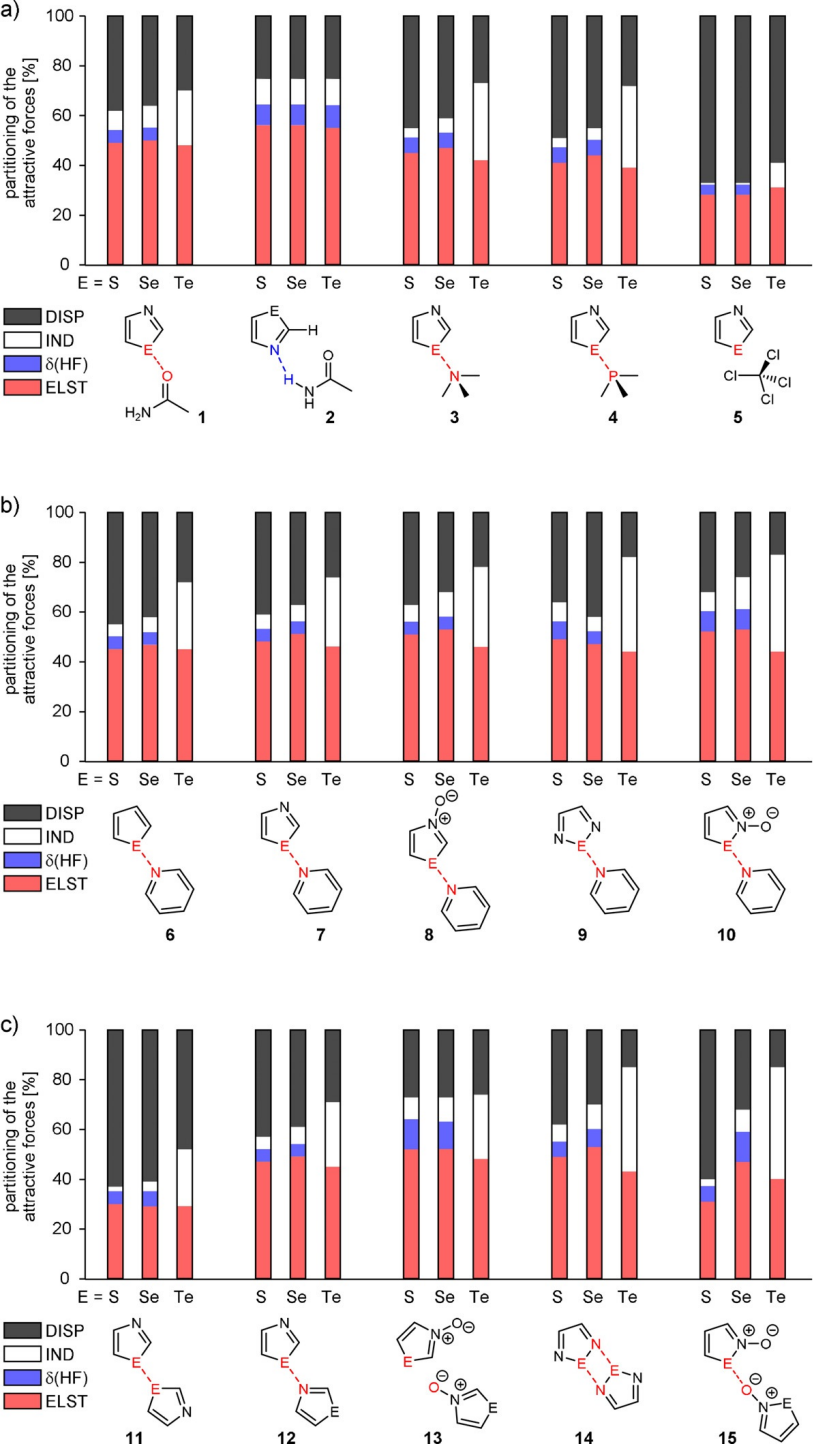
Percentage contributions of the attractive parts (dispersion ($E_{DISP}^{\left(2\right)}$ ), induction ($E_{IND}^{\left(2\right)}$ ) and electrostatic ($E_{ELST}^{\left(1\right)}$ ) term as well as high order corrections $\delta \left(HF\right)$ ) to total attraction ($E^{SAPT}$ ) as derived by the DFT‐SAPT program for model systems **1**–**5** (a), **6**–**10** (b) and **11**–**15** (c).

The data of the SAPT interaction energies and the values of the individual terms for complexes **1**–**15** are summarized in Tables S3 and S4. Additionally, the percentage contributions of the attractive parts (dispersion ($E_{DISP}^{\left(2\right)}$ ), induction ($E_{IND}^{\left(2\right)}$ ), electrostatic ($E_{ELST}^{\left(1\right)}$ ) and $\delta \left(HF\right)$ term) of total attraction are depicted in Figure 2. A comparison shows that the dispersion energy of van der Waals complexes **5 a**–**c**, **11 a** and **15 a** represents the main attraction force. In complexes with weak chalcogen bonds (E=S, Se) the dispersion and the electrostatic term are dominant. In general, the latter is slightly larger. However, the induction term plays a subordinate role and only contributes 3–12 % to the net attractive interactions.

A completely different picture is found for strong chalcogen bonds (E=Te). In these bonds the induction term plays a highly important role and the following trend can be observed: the higher the complex formation energy is, the higher is the percentage of induction energy contributing to the net attractive interactions. Examples underlining this statement are complexes **14 c** (Δ*E*=−15.3 kcal mol^−1^ using CCSD(T) approximation) and **15 c** (CCSD(T): Δ*E*=−20.0 kcal mol^−1^). The percentage of $E_{IND}^{\left(2\right)}$ amounts to 41 % for **14 c** and to 46 % for **15 c**. The latter is the largest term among the attractive interactions. In all other cases of strong chalcogen bonds the electrostatic term is most dominant.

Please note that the dispersion term becomes less important in strong interactions even though tellurium (which can develop extra strong dispersion interactions based on its polarizability) is the element that is involved into chalcogen bonding. Nevertheless, not only dispersion energies in chalcogen bonds increase from sulfur to tellurium but also exchange energies (Pauli repulsion) as well as induction and electrostatic terms. Due to the fact that the latter increase more from S to Te than $E_{DISP}^{\left(2\right)}$ , the significance of dispersion energy drops from S to Te. In case of the strongest chalcogen bonds (**14 c** and **15 c**) the dispersion energy amounts to only 15 %. This is even less than the percentage of dispersion interactions in hydrogen bonds of acetic acid dimers (17 %; see Table S4). A comparison with complexes **2 a**–**c** and **13 a**,**b**, which are formed by hydrogen bonds, is as well interesting. In these complexes the dispersion energy amounts to approximately one quarter of the overall attractive forces. This shows that the dispersion energies of strong chalcogen bonds are—with regard to the attractive forces—on a similar significance level as the dispersion energies of hydrogen bonds.

### Origin of the Chalcogen Bond Orientation

**Figure 3:**
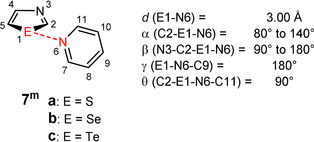
Model **7^m^** to determine the origin for spatial chalcogen bond orientation.

It is well accepted that the relative orientation of atoms being involved in chalcogen bonding can be explained by electrostatic and orbital mixing effects.[^14^, ^34^, ^35^, ^36^] The latter effect is intuitively similar to the DFT‐SAPT induction term. We intended to find out which term is more important for spatial orientation in weak and strong chalcogen bonds, respectively. Therefore, we designed model system **7^m^** (Figure 3) consisting of a chalcogenazole and pyridine. The geometrical parameters of chalcogenazole and pyridine were obtained from B2PLYP‐D3 optimization calculations of the monomers. The distance between E1 and N6 was fixed to a value of 3 Å, and the atoms E1, N6 and C9 were supposed to be in a straight line (*γ*=180°). Additionally, the planes of the aromatic rings had to be perpendicular to one another (*θ*=90°). To determine the origin for chalcogen bond orientation the angles α and β were varied from 80° to 140° and 90° to 180°, respectively.

**Figure 4:**
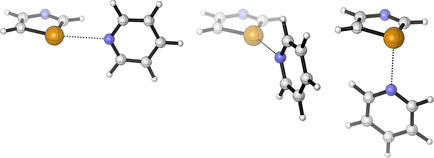
Three conformations of model **7^m^c** to determine the origin of chalcogen bond orientation: conformation **7^m^c‐I** (left; *α*=80°, *β*=180°) corresponds to the geometry of the optimized complexes **7**. The conformations **7^m^c‐II** (center; *α*=140°, *β*=180°) and **7^m^c‐III** (right; *α*=80°, *β*=90°) illustrate highly unfavorable chalcogen bond geometries.

If α is fixed to 80° and β amounts to 180° in this model, conformation **7^m^‐I** (*α*=80°, *β*=180°) is obtained which is very similar to the optimized geometries of **7**: the nitrogen atom of the pyridine points into the area with the most positive electrostatic potential of the chalcogenazole and the n(N) → σ*(E‐R) interaction should be at its highest point in this position (Figure 4 and S46). If α is changed to 140°, conformation **7^m^‐II** (*α*=140°, *β*=180°) is obtained in which the nitrogen atom is oriented centrally toward the chalcogenazole ring (Figure 4). At this position, the lowest positive electrostatic potential inside this plane should be found for the chalcogen atom (Figure S46). The n(N) → σ*(E‐R) interactions should as well be weaker. If α is left at 80° and β is changed to 90°, conformation **7^m^‐III** (*α*=80°, *β*=90°) is obtained in which the nitrogen atom is perpendicular to the chalcogenazole ring (Figure 4). In this case no n(N) → σ*(E‐R) interaction is expected. This position should exhibit the lowest positive electrostatic potential at the chalcogen atom (Figure S46).

Complex formation energies and individual values of the SAPT terms are listed in Tables S5‐S7. The SAPT energies of conformers **I** (*α*=80°, *β*=180°) are only slightly different to the energies of complexes **7** ($E^{SAPT}$ (**7^m^‐I**)‐$\;E^{SAPT}$ (**7**)=0.68 kcal mol^−1^ for S; 0.31 kcal mol^−1^ for Se; 0.20 kcal mol^−1^ for Te). Therefore, conformers **I** of **7^m^** represent a legitimate model for **7**. As expected, conformations **II** and **III** are most unfavorable. An analysis of the single terms of conformations **I**, **II** and **III** should shed light on the origin of chalcogen bond orientation.

**Figure 5:**
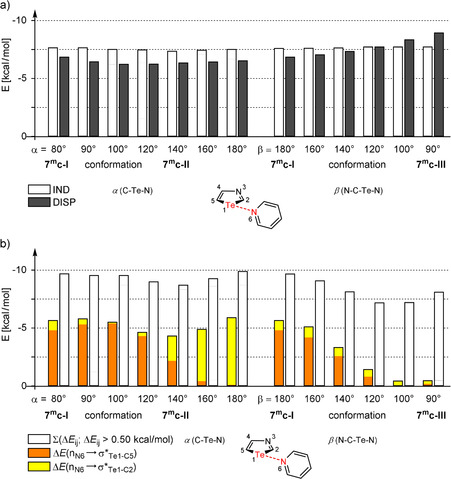
a) Induction ($E_{IND}^{\left(2\right)}$ ) and dispersion ($E_{DISP}^{\left(2\right)}$ ) interactions in model system **7^m^c** depending on angles α and β as derived by the DFT‐SAPT program. b) Stabilization energies Δ*E* between “filled” (donor) NBOs and “empty” (acceptor) NBOs in model system **7^m^c** depending on angles α and β as derived by the NBO program. Beside the two n(N6) → σ*(Te1‐C2/5) interactions the sum of all interaction energies between the two monomers amounting to more than 0.5 kcal mol^−1^ are listed.

At first, we had a look at the induction term ($E_{IND}^{\left(2\right)}$ ) of model system **7^m^c** depending on the angles α and β. Due to the fact that the induction term for tellurium compounds is remarkably larger than the induction term for S and Se compounds (see above), the dependency on the angle should be most obvious. Surprisingly, the induction term only changes slightly with regard to the angles α and β (Figure 5 a). The difference for $E_{IND}^{\left(2\right)}$ between the conformations **I** and **II** amounts to only 0.3 kcal mol^−1^. If conformations **I** and **III** are compared, the induction contribution increases by 0.1 kcal mol^−1^. As conformations **II** and **III** are less favorable (5.1 kcal mol^−1^ and 7.7 kcal mol^−1^, respectively) than **I**, the induction term is almost irrelevant for the orientation of strong chalcogen bonds. The change of dispersion energy with the angles α and β cannot explain the origin of chalcogen bond orientation in **7 c**, either (Figure 5 a).

Since the induction term of the SAPT calculations is related to orbital mixing, NBO (natural bond orbitals) analyses^[53]^ of different geometries of **7^m^c** were carried out to confirm the results. Therefore, stabilization energies Δ*E* between “filled” (donor) NBOs and “empty” (acceptor) NBOs depending on the angles α and β were focused. Beside stabilization energies for the two n(N6) → σ*(Te1‐C2/5) interactions, the sum of all interaction energies between the two monomers showing values higher than 0.5 kcal mol^−1^ is shown in Figure 5 b. Although the whole stabilization energy between the monomers based on orbital mixing is not captured by this cutoff of 0.5 kcal mol^−1^, a trend should be observed. In fact, the n(N6) → σ*(Te1‐C5) interaction decreases by changing the angles α or β. It is however replaced by other donor NBOs → acceptor NBOs so that only a slight change of the sum of stabilization energies based on orbital mixing occurs.

**Figure 6:**
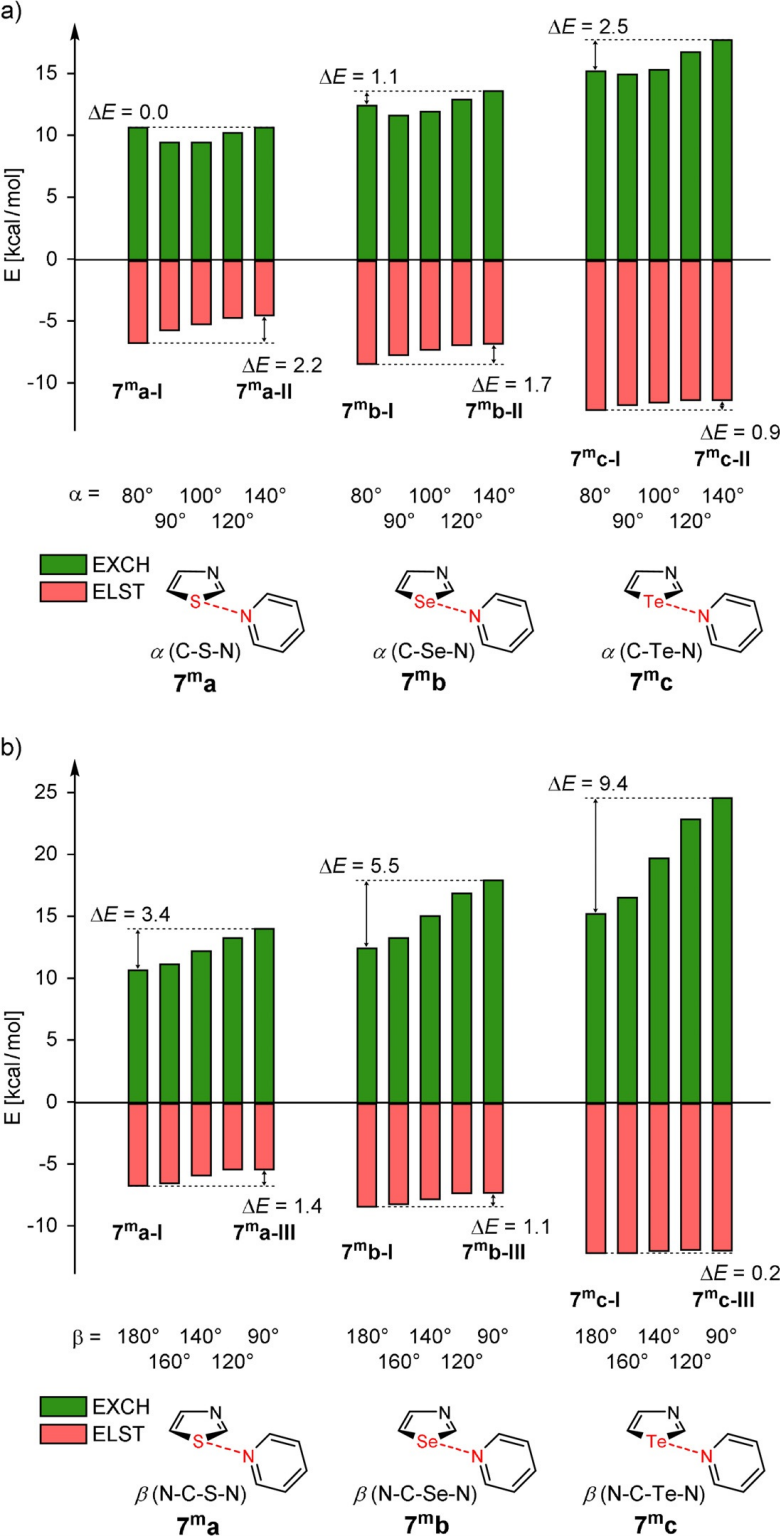
Electrostatic ($E_{ELST}^{\left(1\right)}$ ) and exchange ($E_{EXCH}^{\left(1\right)}$ ) interactions in model systems **7^m^** depending on the angles α (a) and β (b) as derived by the DFT‐SAPT program.

Due to the fact that neither the induction nor the dispersion term could explain the energy changes caused by the geometry of the chalcogen bond in **7 c**, the two other terms are considered in the following. For this purpose, the electrostatic ($E_{ELST}^{\left(1\right)}$ ) and exchange ($E_{EXCH}^{\left(1\right)}$ ) interactions in model systems **7^m^** depending on the angles α and β are illustrated in Figure 6.

First, we consider the changes caused by modifying angle α (conformations **I** and **II** in Figure 6 a). In this case an obvious difference between weak (E=S, Se) and strong (E=Te) chalcogen bonds can be observed. For weak chalcogen bonds the change of the electrostatic interaction term is dominating; the change of the exchange term is distinctly smaller (E=Se) or does not exist (E=S). Thus, the driving force for chalcogen bond orientation is the electrostatic interaction. In case of strong chalcogen bonds (E=Te) the change of the exchange term during transition from conformation **I** to **II** is almost three times as high as the change of the electrostatic term and therefore the dominating part (Figure 6 a). An analogous phenomenon has already been found in halogen bonding: The directionality of halogen bonds has been ascribed to Pauli repulsion.[^54^, ^55^] Far more drastic changes can be observed if conformations **I** and **III** of **7^m^c** are considered (change caused by varying angle β“; Figure 6 b). In this case the change of the exchange term amounts to 9.4 kcal mol^−1^ and the change of the electrostatic term is only 0.2 kcal mol^−1^. As conformation **III** of **7^m^c** (−28.4 kcal mol^−1^ see Table S7) is equipped with stronger net attractive interactions than **I** (−26.5 kcal mol^−1^) and **II** (−24.8 kcal mol^−1^), it can be stated that the Pauli repulsion (steric interaction) disables the formation of conformations **II** and **III** and is therefore responsible for the orientation of the strong chalcogen bond in **7 c**. The analogous models **8^m^c**, **10^m^c**, **12^m^c** and **15^m^c** confirm the dominance of steric interactions for spatial orientation of strong chalcogen bonds in **8 c**, **10 c**, **12 c** and **15 c** (Tables S8‐S11). Please note, a transition from conformation **I** in model **15** ^**m**^ **c** to both conformation **II** and **III** leads to an increase of the electrostatic energy (Table S8).

Using the above described model, the origin of the non‐linearity of chalcogen bonds^[37]^ can be explained. For example, the angles C5‐E1‐N6 in the complexes **7 a**–**c** distinctly deviate from 180° and amount to 161°, 161° and 156°, respectively. A comparison of the four terms $E_{ELST}^{\left(1\right)}$ , $\;E_{EXCH}^{\left(1\right)}$ , $E_{IND}^{\left(2\right)}$ , and $E_{DISP}^{\left(2\right)}$ in the model system **7^m^a–c** shows that the electrostatic and dispersion interactions are the driving forces for the deviation from linearity.

## Conclusion

The nature of chalcogen bonds between chalcogen‐containing aromatics and different Lewis bases was investigated by means of SAPT calculations. We were able to show that the nature of chalcogen bonds in sulfur‐ and selenium‐containing compounds is distinctly different compared to tellurium‐containing compounds. Sulfur and selenium chalcogen bonds are weak and dominated by electrostatic and dispersion interactions. As expected, chalcogen bonds of tellurium‐containing compounds are remarkably stronger. In this case, the attractive interactions are dominated by the electrostatic and the induction term. In contrast to many previous examples the induction term, which corresponds to the orbital mixing contribution, is not crucial for chalcogen bond orientation. Whereas the electrostatic interaction is responsible for orientation in sulfur‐containing systems, the assembly of chalcogen bond atoms in tellurium‐containing compounds is directed by steric interactions.

## Conflict of interest

The authors declare no conflict of interest.

## Supporting information

As a service to our authors and readers, this journal provides supporting information supplied by the authors. Such materials are peer reviewed and may be re‐organized for online delivery, but are not copy‐edited or typeset. Technical support issues arising from supporting information (other than missing files) should be addressed to the authors.

Supplementary
