# Supplementary material for: The Nature of Strong Chalcogen Bonds Involving Chalcogen‐Containing Heterocycles
Source: Angew Chem Int Ed Engl. 2020 Sep 7;59(47):21236–43. doi: 10.1002/anie.202010309 (PMC7693109; doi:10.1002/anie.202010309)
Supplement: Supplementary file 1 — Supplementary [file ANIE-59-21236-s001.pdf]

Angewandte Chemie
Eine Zeitschrift der Gesellschaft Deutscher Chemiker

Supporting Information

The Nature of Strong Chalcogen Bonds Involving Chalcogen-Containing Heterocycles

Gebhard Haberhauer and Rolf Gleiter**

anie_202010309_sm_miscellaneous_information.pdf

Supporting Information

1. **Figures and Tables** S2
2. **Computational Details** S28
3. **Cartesian Coordinates and Absolute Energies for All Calculated Compounds** S30
4. **Supporting Information References** S53

1. Figures and Tables

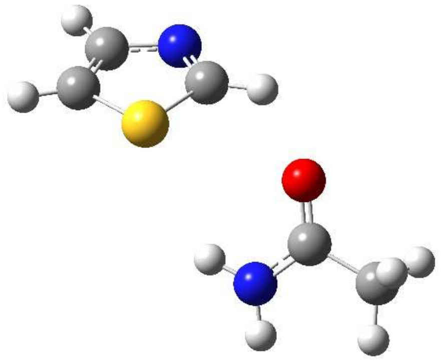

Figure S1. Molecular structure of **1a** calculated by means of B2PLYP-D3/TZVP,aug-cc-pVTZ-PP.

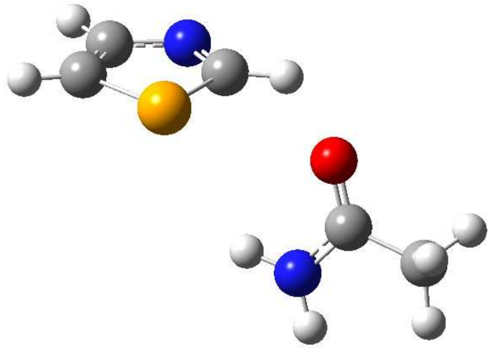

Figure S2. Molecular structure of **1b** calculated by means of B2PLYP-D3/TZVP,aug-cc-pVTZ-PP.

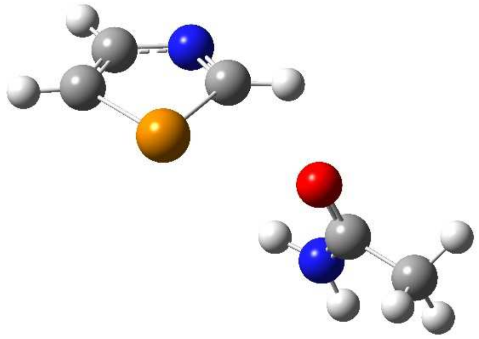

Figure S3. Molecular structure of **1c** calculated by means of B2PLYP-D3/TZVP,aug-cc-pVTZ-PP.

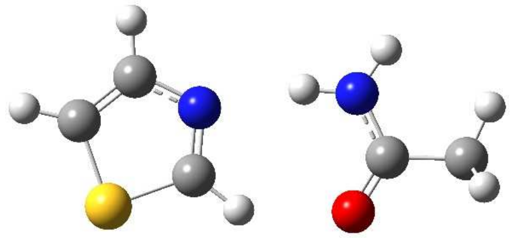

Figure S4. Molecular structure of **2a** calculated by means of B2PLYP-D3/TZVP,aug-cc-pVTZ-PP.

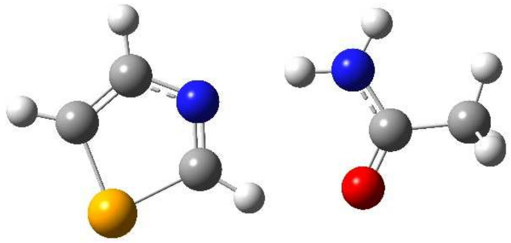

Figure S5. Molecular structure of **2b** calculated by means of B2PLYP-D3/TZVP,aug-cc-pVTZ-PP.

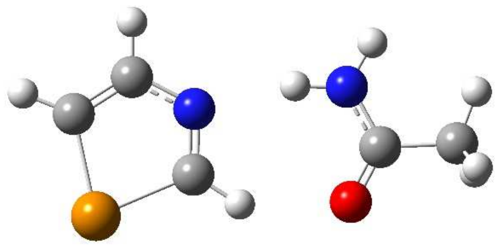

Figure S6. Molecular structure of **2c** calculated by means of B2PLYP-D3/TZVP,aug-cc-pVTZ-PP.

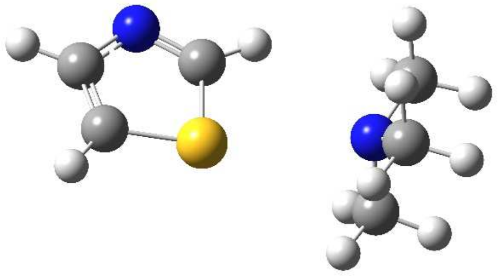

Figure S7. Molecular structure of **3a** calculated by means of B2PLYP-D3/TZVP,aug-cc-pVTZ-PP.

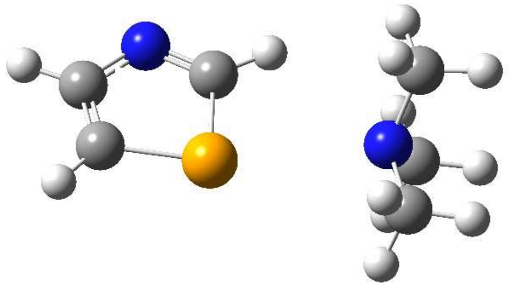

Figure S8. Molecular structure of **3b** calculated by means of B2PLYP-D3/TZVP,aug-cc-pVTZ-PP.

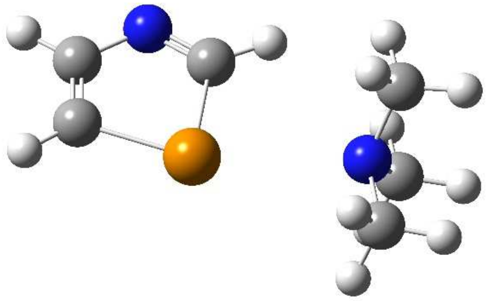

Figure S9. Molecular structure of **3c** calculated by means of B2PLYP-D3/TZVP,aug-cc-pVTZ-PP.

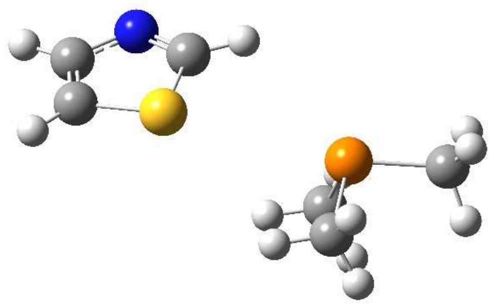

Figure S10. Molecular structure of **4a** calculated by means of B2PLYP-D3/TZVP,aug-cc-pVTZ-PP.

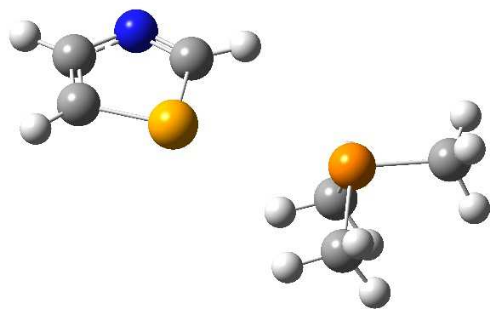

Figure S11. Molecular structure of **4b** calculated by means of B2PLYP-D3/TZVP,aug-cc-pVTZ-PP.

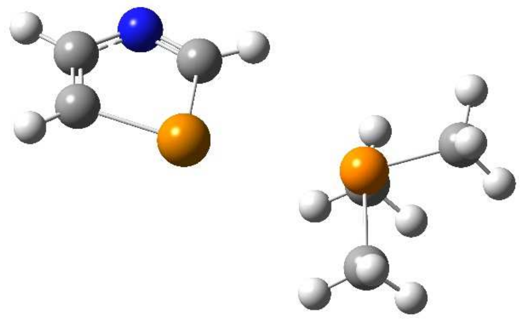

Figure S12. Molecular structure of **4c** calculated by means of B2PLYP-D3/TZVP,aug-cc-pVTZ-PP.

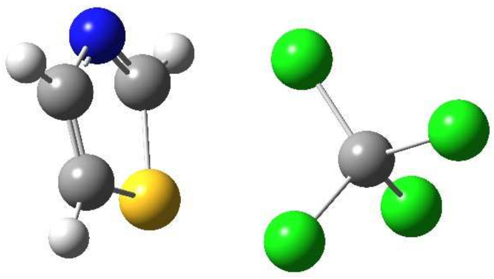

Figure S13. Molecular structure of **5a** calculated by means of B2PLYP-D3/TZVP,aug-cc-pVTZ-PP.

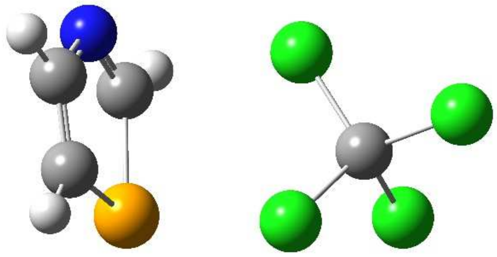

Figure S14. Molecular structure of **5b** calculated by means of B2PLYP-D3/TZVP,aug-cc-pVTZ-PP.

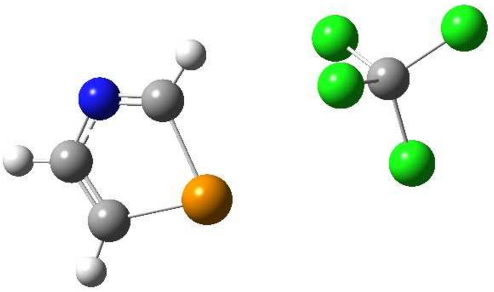

Figure S15. Molecular structure of **5c** calculated by means of B2PLYP-D3/TZVP,aug-cc-pVTZ-PP.

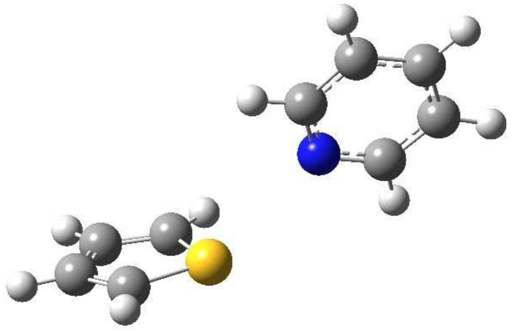

Figure S16. Molecular structure of **6a** calculated by means of B2PLYP-D3/TZVP,aug-cc-pVTZ-PP.

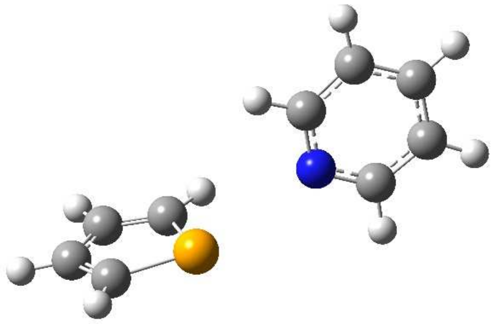

Figure S17. Molecular structure of **6b** calculated by means of B2PLYP-D3/TZVP,aug-cc-pVTZ-PP.

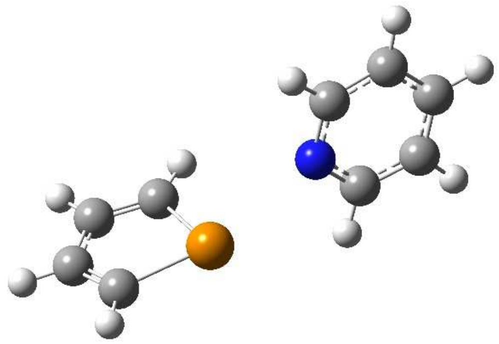

Figure S18. Molecular structure of **6c** calculated by means of B2PLYP-D3/TZVP,aug-cc-pVTZ-PP.

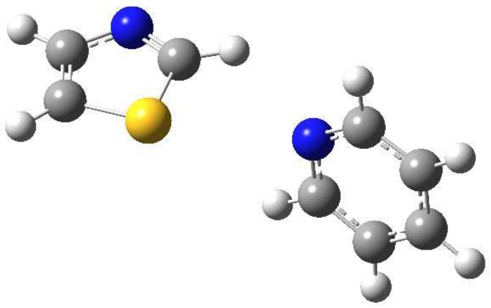

Figure S19. Molecular structure of **7a** calculated by means of B2PLYP-D3/TZVP,aug-cc-pVTZ-PP.

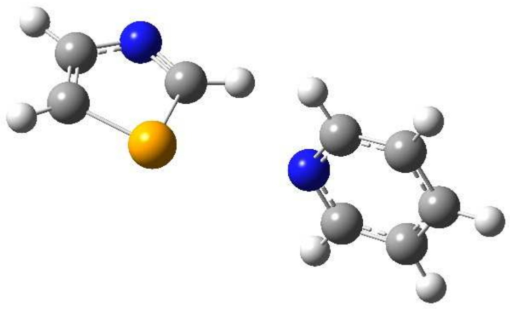

Figure S20. Molecular structure of **7b** calculated by means of B2PLYP-D3/TZVP,aug-cc-pVTZ-PP.

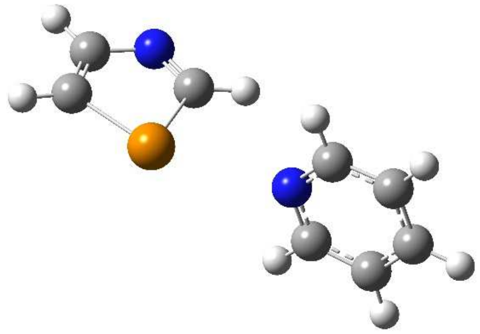

Figure S21. Molecular structure of **7c** calculated by means of B2PLYP-D3/TZVP,aug-cc-pVTZ-PP.

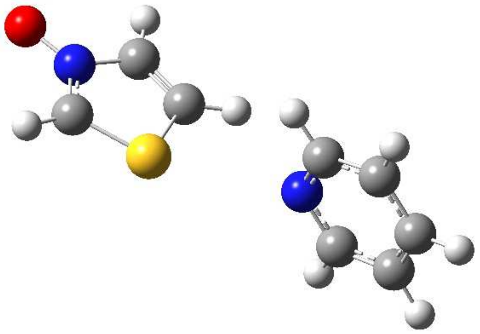

Figure S22. Molecular structure of **8a** calculated by means of B2PLYP-D3/TZVP,aug-cc-pVTZ-PP.

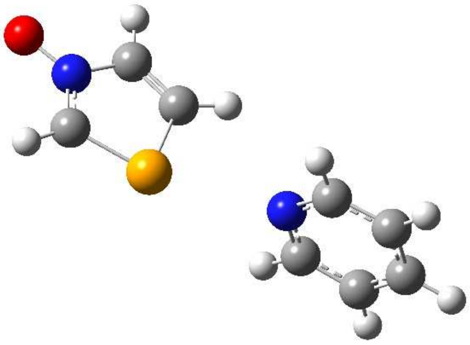

Figure S23. Molecular structure of **8b** calculated by means of B2PLYP-D3/TZVP,aug-cc-pVTZ-PP.

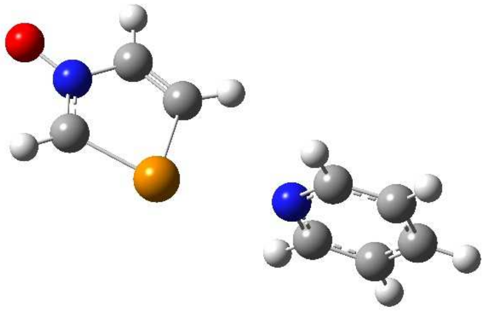

Figure S24. Molecular structure of **8c** calculated by means of B2PLYP-D3/TZVP,aug-cc-pVTZ-PP.

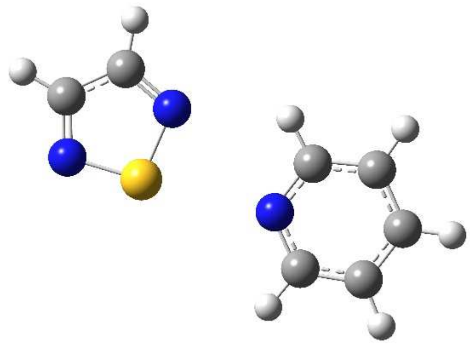

Figure S25. Molecular structure of **9a** calculated by means of B2PLYP-D3/TZVP,aug-cc-pVTZ-PP.

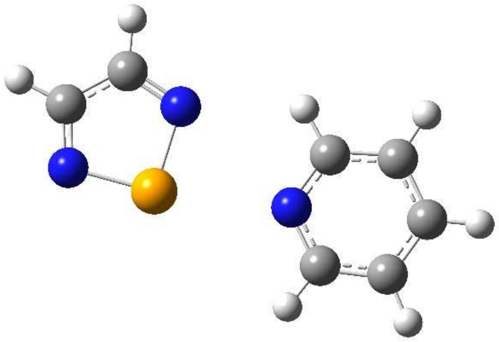

Figure S26. Molecular structure of **9b** calculated by means of B2PLYP-D3/TZVP,aug-cc-pVTZ-PP.

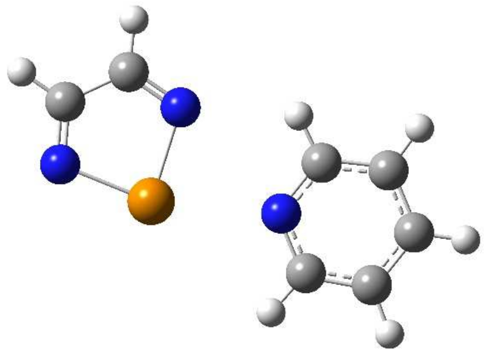

Figure S27. Molecular structure of **9c** calculated by means of B2PLYP-D3/TZVP,aug-cc-pVTZ-PP.

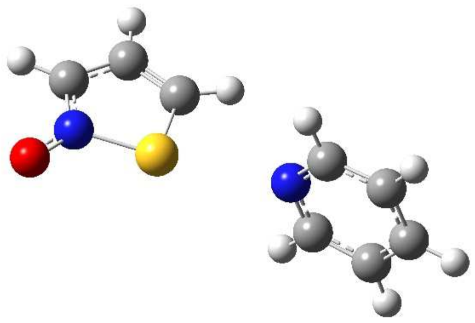

Figure S28. Molecular structure of **10a** calculated by means of B2PLYP-D3/TZVP,aug-cc-pVTZ-PP.

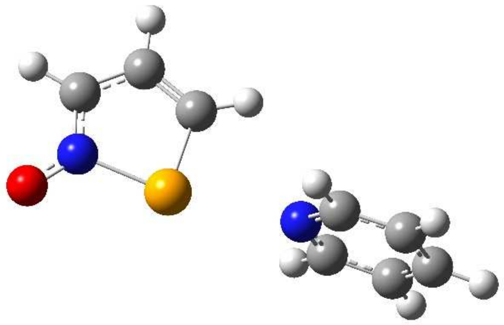

Figure S29. Molecular structure of **10b** calculated by means of B2PLYP-D3/TZVP,aug-cc-pVTZ-PP.

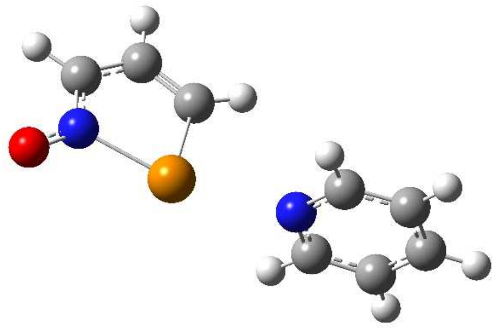

Figure S30. Molecular structure of **10c** calculated by means of B2PLYP-D3/TZVP,aug-cc-pVTZ-PP.

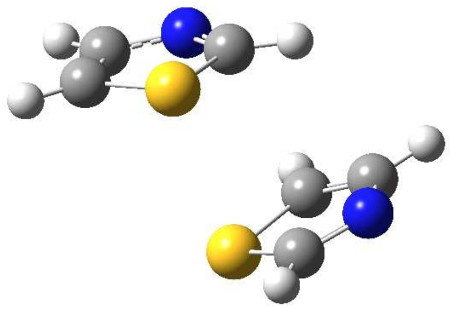

Figure S31. Molecular structure of **11a** calculated by means of B2PLYP-D3/TZVP,aug-cc-pVTZ-PP.

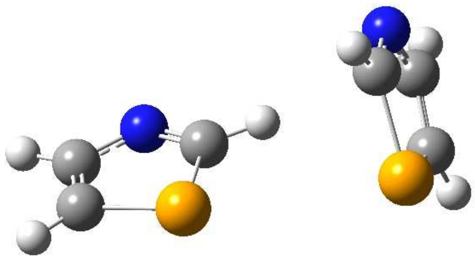

Figure S32. Molecular structure of **11b** calculated by means of B2PLYP-D3/TZVP,aug-cc-pVTZ-PP.

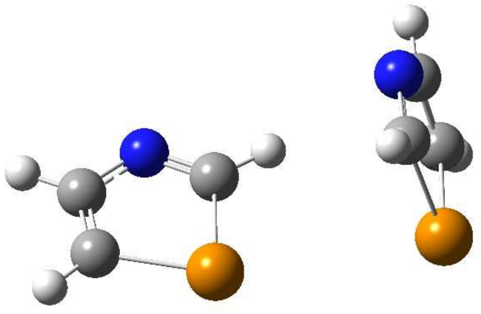

Figure S33. Molecular structure of **11c** calculated by means of B2PLYP-D3/TZVP,aug-cc-pVTZ-PP.

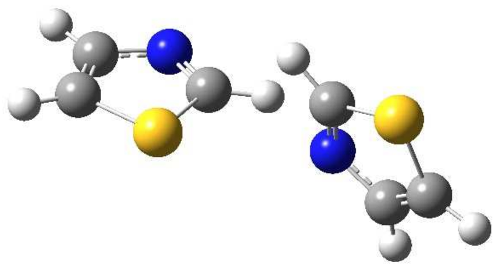

Figure S34. Molecular structure of **12a** calculated by means of B2PLYP-D3/TZVP,aug-cc-pVTZ-PP.

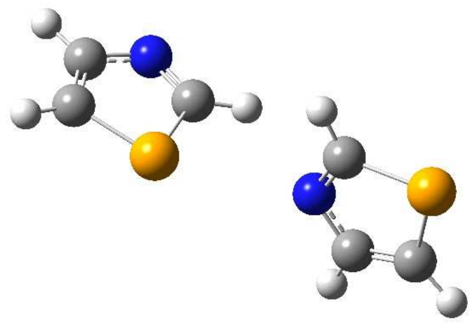

Figure S35. Molecular structure of **12b** calculated by means of B2PLYP-D3/TZVP,aug-cc-pVTZ-PP.

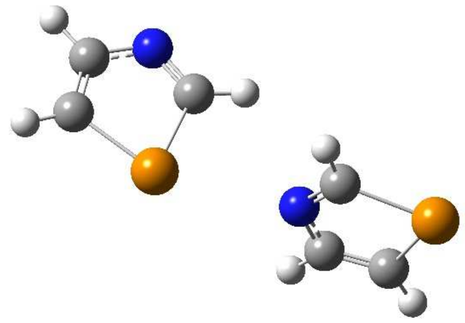

Figure S36. Molecular structure of **12c** calculated by means of B2PLYP-D3/TZVP,aug-cc-pVTZ-PP.

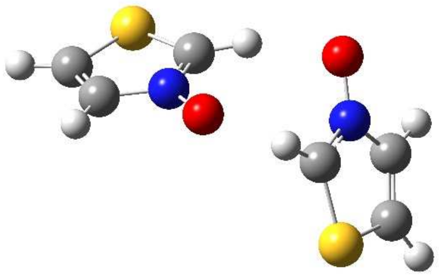

Figure S37. Molecular structure of **13a** calculated by means of B2PLYP-D3/TZVP,aug-cc-pVTZ-PP.

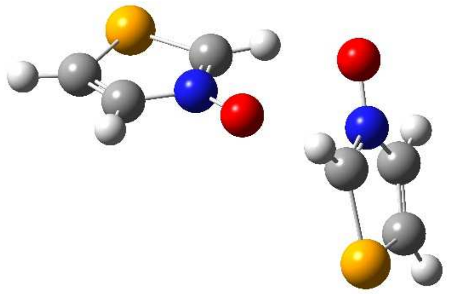

Figure S38. Molecular structure of **13b** calculated by means of B2PLYP-D3/TZVP,aug-cc-pVTZ-PP.

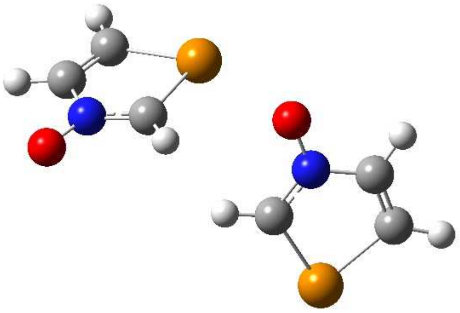

Figure S39. Molecular structure of **13c** calculated by means of B2PLYP-D3/TZVP,aug-cc-pVTZ-PP.

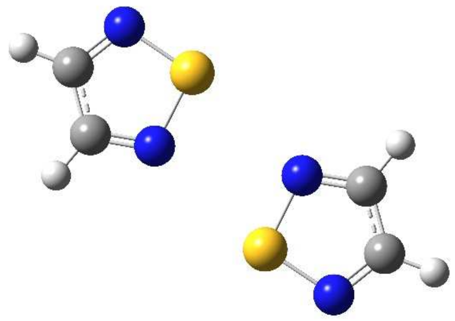

Figure S40. Molecular structure of **14a** calculated by means of B2PLYP-D3/TZVP,aug-cc-pVTZ-PP.

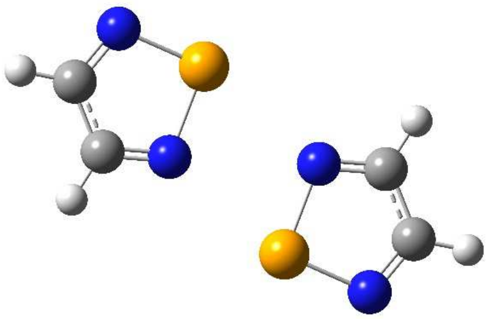

Figure S41. Molecular structure of **14b** calculated by means of B2PLYP-D3/TZVP,aug-cc-pVTZ-PP.

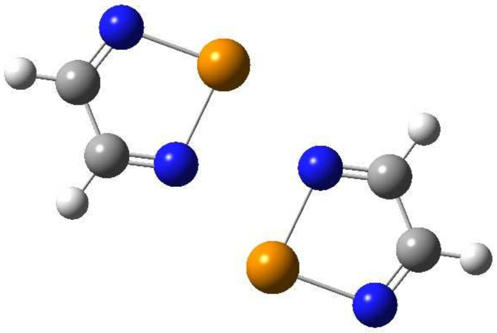

Figure S42. Molecular structure of **14c** calculated by means of B2PLYP-D3/TZVP,aug-cc-pVTZ-PP.

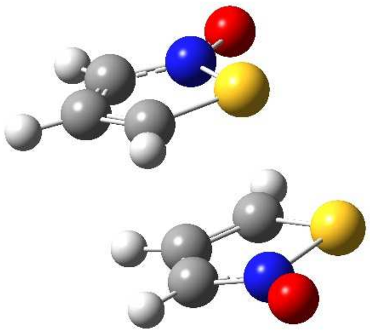

Figure S43. Molecular structure of **15a** calculated by means of B2PLYP-D3/TZVP,aug-cc-pVTZ-PP.

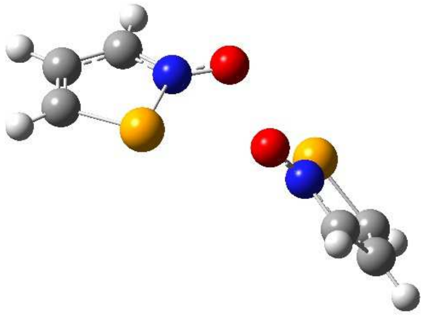

Figure S44. Molecular structure of **15b** calculated by means of B2PLYP-D3/TZVP,aug-cc-pVTZ-PP.

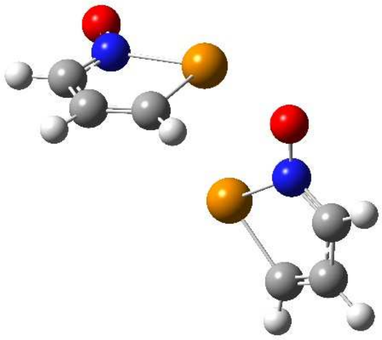

Figure S45. Molecular structure of **15c** calculated by means of B2PLYP-D3/TZVP,aug-cc-pVTZ-PP.

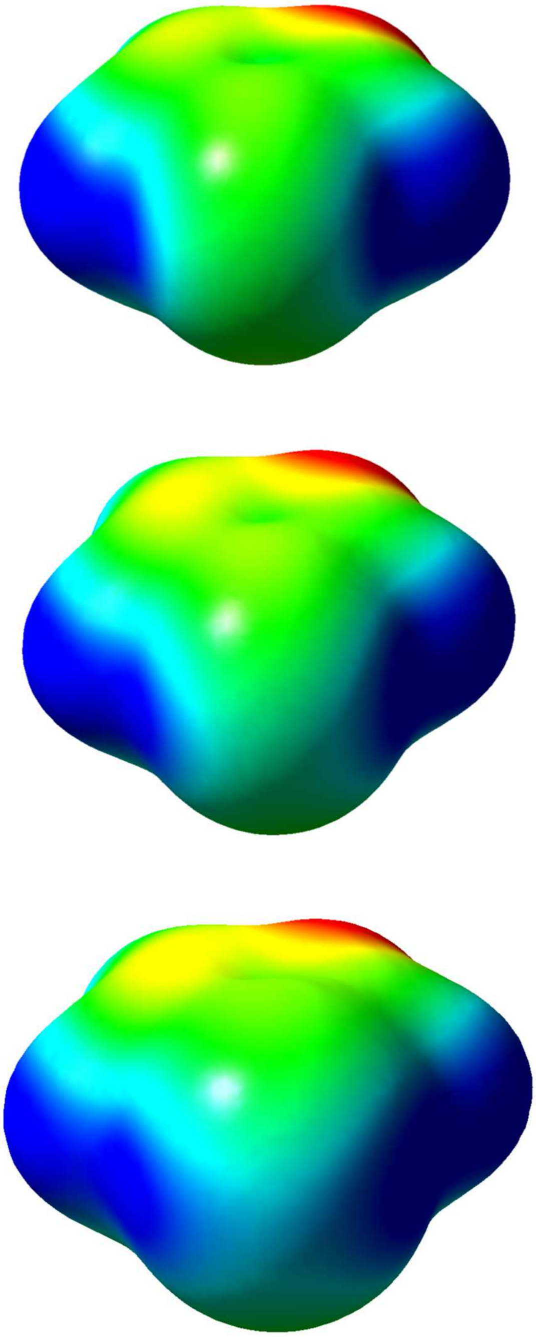

Figure S46. Calculated (B3LYP) molecular electrostatic potentials on the 0.001 au electron density isosurface of 1,3-thiazole (top), 1,3-selenazole (middle) and 1,3-tellurazole (bottom). Color scheme spanning between -0.03 (red) and +0.03 (blue).

Table S1. Formation energies Δ*E* of the complexes **1-8** calculated by means of different methods.

Complex	B2PLYP-D3[a]	B2PLYP[b]	B3LYP-D3BJ[c]	B3LYP[d]	CCSD(T)[e]
**1a**	-5.07	-3.69	-5.47	-2.74	-4.74
**1b**	-5.73	-4.09	-6.25	-3.02	-5.28
**1c**	-7.53	-5.44	-7.81	-3.75	-7.64
**2a**	-9.89	-8.59	-10.45	-7.80	-9.09
**2b**	-9.81	-8.48	-10.37	-7.67	-9.02
**2c**	-9.63	-8.27	-10.19	-7.44	-8.85
**3a**	-5.11	-2.97	-5.47	-1.27	-4.90
**3b**	-6.06	-3.51	-6.66	-1.65	-5.57
**3c**	-8.69	-5.42	-8.92	-2.61	-9.14
**4a**	-3.77	-1.77	-4.18	-0.37	-3.33
**4b**	-4.32	-2.10	-4.84	-0.68	-3.65
**4c**	-6.26	-3.53	-6.40	-1.39	-6.40
**5a**	-3.24	-0.62	-3.62	1.39	-2.95
**5b**	-3.37	-0.59	-3.80	1.49	-2.96
**5c**	-4.15	-1.38	-4.02	1.17	-4.96
**6a**	-3.99	-2.51	-4.12	-1.23	-3.88
**6b**	-4.56	-2.79	-4.74	-1.32	-4.32
**6c**	-6.51	-4.28	-6.54	-2.29	-6.64
**7a**	-4.71	-3.16	-4.85	-1.82	-4.52
**7b**	-5.54	-3.75	-5.81	-2.36	-5.11
**7c**	-7.57	-5.28	-7.65	-3.29	-7.60
**8a**	-6.02	-4.39	-6.17	-2.97	-5.81
**8b**	-7.02	-5.15	-7.36	-3.72	-6.51
**8c**	-9.56	-7.14	-9.67	-5.05	-9.58

[a] B2PLYP-D3/TZVP,aug-cc-pVTZ-PP

[b] B2PLYP/TZVP,aug-cc-pVTZ//B2PLYP-D3/TZVP,aug-cc-pVTZ-PP

[c] B3LYP-D3BJ/TZVP,aug-cc-pVTZ//B2PLYP-D3/TZVP,aug-cc-pVTZ-PP

[d] B3LYP/TZVP,aug-cc-pVTZ//B2PLYP-D3/TZVP,aug-cc-pVTZ-PP

[e] CCSD(T)/TZVP,aug-cc-pVTZ//B2PLYP-D3/TZVP,aug-cc-pVTZ-PP

Table S2. Formation energies ΔE of the complexes **9-15** and the acetic acid dimer calculated by means of different methods.

Complex	B2PLYP-D3[a]	B2PLYP[b]	B3LYP-D3BJ[c]	B3LYP[d]	CCSD(T)[e]
**9a**	-4.68	-3.12	-5.02	-1.96	-4.00
**9b**	-7.11	-5.13	-7.79	-3.92	-5.80
**9c**	-11.90	-9.27	-12.25	-7.20	-11.16
**10a**	-6.53	-4.75	-6.83	-3.34	-6.19
**10b**	-11.42	-9.26	-12.09	-7.91	-8.33
**10c**	-12.63	-9.92	-12.62	-7.46	-12.91
**11a**	-4.35	-1.37	-4.40	1.35	-4.46
**11b**	-3.76	-1.31	-4.09	0.57	-3.49
**11c**	-5.17	-1.88	-5.16	0.83	-5.37
**12a**	-4.37	-2.91	-4.46	-1.63	-4.21
**12b**	-5.11	-3.40	-5.29	-1.97	-4.78
**12c**	-6.92	-4.65	-7.00	-2.68	-6.76
**13a**	-12.25	-10.39	-12.57	-8.87	-12.52
**13b**	-11.97	-10.02	-12.41	-8.51	-12.08
**13c**	-11.67	-8.94	-11.60	-6.34	-12.15
**14a**	-4.56	-2.87	-4.87	-1.56	-3.78
**14b**	-8.09	-5.67	-8.82	-4.18	-6.30
**14c**	-17.85	-13.95	-18.64	-11.54	-15.27
**15a**	-6.96	-2.92	-6.89	0.95	-7.44
**15b**	-7.70	-5.30	-8.30	-3.65	-6.83
**15c**	-18.81	-14.35	-18.83	-10.89	-20.04
AcOH dimer	-17.52	-16.28	-18.84	-16.24	-15.48

[a] B2PLYP-D3/TZVP,aug-cc-pVTZ-PP

[b] B2PLYP/TZVP,aug-cc-pVTZ//B2PLYP-D3/TZVP,aug-cc-pVTZ-PP

[c] B3LYP-D3BJ/TZVP,aug-cc-pVTZ//B2PLYP-D3/TZVP,aug-cc-pVTZ-PP

[d] B3LYP/TZVP,aug-cc-pVTZ//B2PLYP-D3/TZVP,aug-cc-pVTZ-PP

[e] CCSD(T)/TZVP,aug-cc-pVTZ//B2PLYP-D3/TZVP,aug-cc-pVTZ-PP

Table S3. Contribution of the electrostatic ($E^{(1)}_{ELST}$), exchange ($E^{(1)}_{EXCH}$), induction ($E^{(2)}_{IND} = E^{(2)}_{ind} + E^{(2)}_{exch-ind}$), dispersion ($E^{(2)}_{DISP} = E^{(2)}_{disp} + E^{(2)}_{exch-disp}$) energies and $\delta(HF)$ term to the interaction energy E^{SAPT} in the complexes **1-8**. All values are given in kcal/mol.

complex	$E^{(1)}_{ELST}$	$E^{(1)}_{EXCH}$	$E^{(2)}_{IND}$	$E^{(2)}_{DISP}$	$\delta(HF)$	E^{SAPT}
**1a**	-5.91	8.03	-1.04	-4.56	-0.65	-4.14
**1b**	-7.22	9.75	-1.26	-5.24	-0.77	-4.74
**1c**	-11.30	14.66	-5.11	-6.93	0.97	-7.71
**2a**	-14.19	17.45	-2.73	-6.20	-2.09	-7.75
**2b**	-14.06	17.51	-2.73	-6.27	-2.11	-7.66
**2c**	-13.77	17.47	-2.73	-6.35	-2.14	-7.52
**3a**	-5.97	9.66	-0.57	-6.06	-0.80	-3.74
**3b**	-8.35	13.43	-1.18	-7.26	-1.09	-4.45
**3c**	-14.87	20.44	-11.31	-9.64	5.92	-9.46
**4a**	-4.11	6.68	-0.33	-4.90	-0.63	-3.29
**4b**	-5.36	8.42	-0.63	-5.49	-0.76	-3.82
**4c**	-10.42	13.68	-8.96	-7.50	7.74	-5.46
**5a**	-2.42	5.94	-0.11	-5.93	-0.34	-2.87
**5b**	-2.55	6.19	-0.12	-6.13	-0.36	-2.97
**5c**	-3.12	6.77	-1.05	-6.03	0.25	-3.18
**6a**	-4.11	6.14	-0.49	-4.12	-0.47	-3.05
**6b**	-5.57	8.52	-0.78	-5.04	-0.58	-3.45
**6c**	-11.06	14.54	-6.72	-6.81	2.96	-7.10
**7a**	-5.37	7.56	-0.65	-4.56	-0.56	-3.57
**7b**	-7.36	10.33	-1.12	-5.31	-0.74	-4.20
**7c**	-12.54	15.76	-7.73	-7.12	3.30	-8.32
**8a**	-6.84	8.87	-0.88	-4.97	-0.69	-4.52
**8b**	-9.56	12.61	-1.62	-5.83	-0.95	-5.35
**8c**	-16.42	19.74	-11.14	-8.00	4.91	-10.91

Table S4. Contribution of the electrostatic ($E^{(1)}_{ELST}$), exchange ($E^{(1)}_{EXCH}$), induction ($E^{(2)}_{IND} = E^{(2)}_{ind} + E^{(2)}_{exch-ind}$), dispersion ($E^{(2)}_{DISP} = E^{(2)}_{disp} + E^{(2)}_{exch-disp}$) energies and $\delta(HF)$ term to the interaction energy E^{SAPT} in the complexes **9-15** and the acetic acid dimer. All values are given in kcal/mol.

complex	$E^{(1)}_{ELST}$	$E^{(1)}_{EXCH}$	$E^{(2)}_{IND}$	$E^{(2)}_{DISP}$	$\delta(HF)$	E^{SAPT}
**9a**	-7.56	12.10	-1.14	-5.57	-1.06	-3.24
**9b**	-5.57	8.52	-0.78	-5.04	-0.58	-3.45
**9c**	-27.29	35.40	-23.01	-11.14	10.17	-15.86
**10a**	-10.47	15.87	-1.70	-6.40	-1.71	-4.41
**10b**	-16.92	25.76	-3.96	-8.35	-2.65	-6.12
**10c**	-30.14	39.18	-27.16	-11.45	12.25	-17.32
**11a**	-3.18	7.06	-0.22	-6.62	-0.48	-3.44
**11b**	-2.53	5.71	-0.37	-5.28	-0.53	-3.00
**11c**	-4.14	7.74	-3.19	-6.84	2.80	-3.63
**12a**	-4.64	6.57	-0.53	-4.20	-0.47	-3.27
**12b**	-6.15	8.79	-0.84	-4.96	-0.63	-3.79
**12c**	-10.43	13.50	-6.11	-6.76	2.61	-7.18
**13a**	-17.27	24.73	-3.25	-8.82	-3.88	-8.49
**13b**	-16.80	24.11	-3.13	-8.88	-3.71	-8.40
**13c**	-18.86	25.39	-10.53	-10.06	3.10	-10.95
**14a**	-7.34	11.99	-0.92	-5.72	-0.89	-2.89
**14b**	-15.75	24.13	-2.99	-8.81	-2.05	-5.47
**14c**	-52.96	71.53	-50.40	-18.49	22.75	-27.57
**15a**	-5.41	11.64	-0.44	-10.23	-1.09	-5.53
**15b**	-15.71	27.77	-2.97	-10.92	-4.04	-5.88
**15c**	-63.43	93.21	-73.47	-22.89	46.12	-20.45
AcOH dimer	-33.61	45.76	-9.56	-10.14	-7.42	-14.97

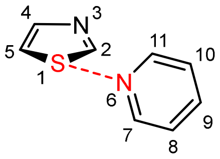

7^m^a

d (S1-N6) = 3.00 Å
α (C2-S1-N6) = 80° to 140°
β (N3-C2-S1-N6) = 90° to 180°
γ (S1-N6-C9) = 180°
θ (C2-S1-N6-C11) = 90°

Table S5. Contribution of the electrostatic ($E^{(1)}_{ELST}$), exchange ($E^{(1)}_{EXCH}$), induction ($E^{(2)}_{IND} = E^{(2)}_{ind} + E^{(2)}_{exch-ind}$), dispersion ($E^{(2)}_{DISP} = E^{(2)}_{disp} + E^{(2)}_{exch-disp}$) energies and $\delta(HF)$ term to the interaction energy E^{SAPT} in the model complexes **7^m^a**. The angles are given in °, the energy values are in kcal/mol. The conformations **I** (α = 80°, β = 180°), **II** (α = 140°, β = 180°) and **III** (α = 80°, β = 90°) are indicated in yellow.

conformation	α	β	$E^{(1)}_{ELST}$	$E^{(1)}_{EXCH}$	$E^{(2)}_{IND}$	$E^{(2)}_{DISP}$	$\delta(HF)$	E^{SAPT}
**7^m^a-I**	80	180	-6.62	10.64	-0.96	-5.15	-0.80	-2.89
	90	180	-5.62	9.39	-0.87	-4.51	-0.75	-2.37
	100	180	-5.11	9.45	-0.81	-4.24	-0.72	-1.43
	120	180	-4.57	10.24	-0.70	-4.10	-0.61	0.25
**7^m^a-II**	140	180	-4.45	10.60	-0.67	-4.11	-0.58	0.80
	80	160	-6.36	11.10	-0.93	-5.17	-0.81	-2.17
	80	140	-5.79	12.20	-0.89	-5.25	-0.80	-0.53
	80	120	-5.27	13.18	-0.87	-5.44	-0.75	0.86
	80	100	-5.05	13.53	-0.89	-5.95	-0.70	0.93
**7^m^a-III**	80	90	-5.26	14.03	-0.92	-6.57	-0.69	0.60

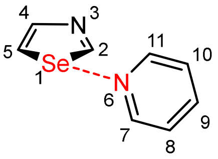

$7^{m}b$

d (Se1-N6) = 3.00 Å
α (C2-Se1-N6) = 80° to 140°
β (N3-C2-Se1-N6) = 90° to 180°
γ (Se1-N6-C9) = 180°
θ (C2-Se1-N6-C11) = 90°

Table S6. Contribution of the electrostatic ($E^{(1)}_{ELST}$), exchange ($E^{(1)}_{EXCH}$), induction ($E^{(2)}_{IND} = E^{(2)}_{ind} + E^{(2)}_{exch-ind}$), dispersion ($E^{(2)}_{DISP} = E^{(2)}_{disp} + E^{(2)}_{exch-disp}$) energies and $\delta(HF)$ term to the interaction energy E^{SAPT} in the model complexes **$7^{m}b$**. The angles are given in °, the energy values are in kcal/mol. The conformations **I** (α = 80°, β = 180°), **II** (α = 140°, β = 180°) and **III** (α = 80°, β = 90°) are indicated in yellow.

conformation	α	β	$E^{(1)}_{ELST}$	$E^{(1)}_{EXCH}$	$E^{(2)}_{IND}$	$E^{(2)}_{DISP}$	$\delta(HF)$	E^{SAPT}
**$7^{m}b$-I**	80	180	-8.32	12.42	-1.44	-5.67	-0.89	-3.89
	90	180	-7.58	11.62	-1.36	-5.11	-0.88	-3.31
	100	180	-7.20	11.87	-1.29	-4.88	-0.85	-2.35
	120	180	-6.78	12.89	-1.17	-4.78	-0.70	-0.54
**$7^{m}b$-II**	140	180	-6.66	13.50	-1.12	-4.82	-0.64	0.25
	80	160	-8.12	13.20	-1.41	-5.73	-0.93	-3.00
	80	140	-7.68	15.05	-1.38	-5.90	-0.95	-0.86
	80	120	-7.25	16.77	-1.37	-6.17	-0.86	1.13
	80	100	-7.03	17.46	-1.39	-6.71	-0.78	1.55
**$7^{m}b$-III**	80	90	-7.20	17.93	-1.41	-7.31	-0.78	1.24

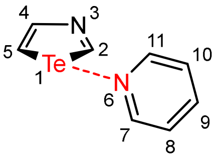

$7^{m}c$

d (Te1-N6) = 3.00 Å
α (C2-Te1-N6) = 80° to 140°
β (N3-C2-Te1-N6) = 90° to 180°
γ (Te1-N6-C9) = 180°
θ (C2-Te1-N6-C11) = 90°

Table S7. Contribution of the electrostatic ($E^{(1)}_{ELST}$), exchange ($E^{(1)}_{EXCH}$), induction ($E^{(2)}_{IND} = E^{(2)}_{ind} + E^{(2)}_{exch-ind}$), dispersion ($E^{(2)}_{DISP} = E^{(2)}_{disp} + E^{(2)}_{exch-disp}$) energies and $\delta(HF)$ term to the interaction energy E^{SAPT} in the model complexes **$7^{m}c$**. The angles are given in °, the energy values are in kcal/mol. The conformations **I** (α = 80°, β = 180°), **II** (α = 140°, β = 180°) and **III** (α = 80°, β = 90°) are indicated in yellow.

conformation	α	β	$E^{(1)}_{ELST}$	$E^{(1)}_{EXCH}$	$E^{(2)}_{IND}$	$E^{(2)}_{DISP}$	$\delta(HF)$	E^{SAPT}
**$7^{m}c$-I**	80	180	-12.05	15.12	-7.63	-6.85	3.29	-8.13
	90	180	-11.63	14.77	-7.59	-6.40	3.36	-7.48
	100	180	-11.42	15.23	-7.53	-6.24	3.53	-6.44
	120	180	-11.19	16.60	-7.40	-6.23	3.96	-4.26
**$7^{m}c$-II**	140	180	-11.16	17.63	-7.34	-6.32	4.18	-3.01
	80	160	-12.01	16.45	-7.62	-6.97	3.16	-6.98
	80	140	-11.93	19.66	-7.62	-7.29	3.13	-4.05
	80	120	-11.83	22.76	-7.67	-7.72	3.45	-1.00
	80	100	-11.74	24.09	-7.70	-8.29	3.61	-0.03
**$7^{m}c$-III**	80	90	-11.87	24.50	-7.70	-8.85	3.52	-0.40

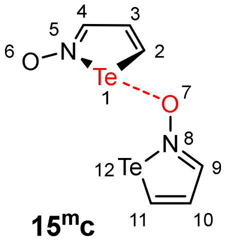

15^mc

d (Te1-O7) = 2.37 Å
α (C2-Te1-O7) = 85° to 140°
β (C3-C2-Te1-N6) = 90° to 180°
γ (Te1-O7-N8) = 107°
θ (C2-Te1-O7-N8) = 90°

Table S8. Contribution of the electrostatic ($E^{(1)}_{ELST}$), exchange ($E^{(1)}_{EXCH}$), induction ($E^{(2)}_{IND} = E^{(2)}_{ind} + E^{(2)}_{exch-ind}$), dispersion ($E^{(2)}_{DISP} = E^{(2)}_{disp} + E^{(2)}_{exch-disp}$) energies and $\delta(HF)$ term to the interaction energy E^{SAPT} in the model complexes **15^mc**. The angles are given in °, the energy values are in kcal/mol. The conformations **I** (α = 85°, β = 180°), **II** (α = 140°, β = 180°) and **III** (α = 85°, β = 90°) are indicated in yellow.

conformation	α	β	$E^{(1)}_{ELST}$	$E^{(1)}_{EXCH}$	$E^{(2)}_{IND}$	$E^{(2)}_{DISP}$	$\delta(HF)$	E^{SAPT}
**15^mc-I**	85	180	-59.45	92.11	-72.36	-22.67	49.45	-12.92
	95	180	-59.86	92.69	-73.15	-22.13	50.49	-11.96
	105	180	-60.46	94.39	-73.89	-21.95	52.17	-9.74
	120	180	-61.00	96.78	-74.55	-21.93	55.42	-5.29
**15^mc-II**	140	180	-60.81	98.13	-74.57	-22.00	59.10	-0.15
	85	160	-54.81	92.95	-64.96	-22.30	40.47	-8.65
	85	140	-52.67	99.53	-58.70	-22.54	32.44	-1.94
	85	120	-53.98	108.20	-57.99	-23.22	34.66	7.67
	85	100	-58.06	114.60	-64.35	-24.09	40.83	8.93
**15^mc-III**	85	90	-60.55	116.21	-69.06	-24.61	44.01	6.01

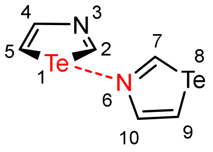

$\mathbf{12^{m}c}$

d (Te1-N6) =	3.00 Å
α (C2-Te1-N6) =	80° to 140°
β (N3-C2-Te1-N6) =	90° to 180°
γ (Te1-N6-Te8) =	160°
θ (C2-Te1-N6-C7) =	90°

Table S9. Contribution of the electrostatic ($E^{(1)}_{ELST}$), exchange ($E^{(1)}_{EXCH}$), induction ($E^{(2)}_{IND} = E^{(2)}_{ind} + E^{(2)}_{exch-ind}$), dispersion ($E^{(2)}_{DISP} = E^{(2)}_{disp} + E^{(2)}_{exch-disp}$) energies and $\delta(HF)$ term to the interaction energy E^{SAPT} in the model complexes $\mathbf{12^{m}c}$. The angles are given in °, the energy values are in kcal/mol.

conformation	α	β	$E^{(1)}_{ELST}$	$E^{(1)}_{EXCH}$	$E^{(2)}_{IND}$	$E^{(2)}_{DISP}$	$\delta(HF)$	E^{SAPT}
$\mathbf{12^{m}c}$**-I**	80	180	-11.05	14.60	-6.94	-6.89	3.15	-7.14
$\mathbf{12^{m}c}$**-II**	140	180	-10.41	17.05	-6.64	-6.36	4.02	-2.34
$\mathbf{12^{m}c}$**-III**	80	90	-11.19	23.59	-6.93	-8.74	3.38	0.11

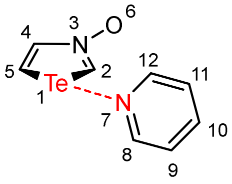

$\mathbf{8^{m}c}$

d (Te1-N7) =	2.90 Å
α (C2-Te1-N7) =	80° to 140°
β (N3-C2-Te1-N7) =	90° to 180°
γ (Te1-N7-C10) =	180°
θ (C2-Te1-N7-C12) =	90°

Table S10. Contribution of the electrostatic ($E^{(1)}_{ELST}$), exchange ($E^{(1)}_{EXCH}$), induction ($E^{(2)}_{IND} = E^{(2)}_{ind} + E^{(2)}_{exch-ind}$), dispersion ($E^{(2)}_{DISP} = E^{(2)}_{disp} + E^{(2)}_{exch-disp}$) energies and $\delta(HF)$ term to the interaction energy E^{SAPT} in the model complexes $\mathbf{8^{m}c}$. The angles are given in °, the energy values are in kcal/mol.

conformation	α	β	$E^{(1)}_{ELST}$	$E^{(1)}_{EXCH}$	$E^{(2)}_{IND}$	$E^{(2)}_{DISP}$	$\delta(HF)$	E^{SAPT}
$\mathbf{8^{m}c}$**-I**	80	180	-16.01	19.60	-11.03	-7.82	4.79	-10.46
$\mathbf{8^{m}c}$**-II**	140	180	-15.08	21.95	-10.62	-7.21	6.29	-4.68
$\mathbf{8^{m}c}$**-III**	80	90	-16.25	31.76	-11.18	-10.35	5.56	-0.46

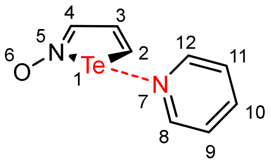

10^m c

d (Te1-N7) = 2.65 Å
α (C2-Te1-N7) = 80° to 140°
β (C3-C2-Te1-N7) = 90° to 180°
γ (Te1-N7-C10) = 180°
θ (C2-Te1-N7-C12) = 90°

Table S11. Contribution of the electrostatic ($E_{ELST}^{(1)}$), exchange ($E_{EXCH}^{(1)}$), induction ($E_{IND}^{(2)} = E_{ind}^{(2)} + E_{exch-ind}^{(2)}$), dispersion ($E_{DISP}^{(2)} = E_{disp}^{(2)} + E_{exch-disp}^{(2)}$) energies and $\delta(HF)$ term to the interaction energy E^{SAPT} in the model complexes **10^m c**. The angles are given in °, the energy values are in kcal/mol.

conformation	α	β	$E_{ELST}^{(1)}$	$E_{EXCH}^{(1)}$	$E_{IND}^{(2)}$	$E_{DISP}^{(2)}$	$\delta(HF)$	E^{SAPT}
**10^m c-I**	80	180	-30.09	40.07	-26.79	-11.71	12.18	-16.35
**10^m c-II**	140	180	-28.94	43.42	-26.74	-10.84	16.99	-6.10
**10^m c-III**	80	90	-33.05	61.93	-29.36	-15.24	18.88	3.15

2. Computational Details

All calculations were performed by using the program packages Gaussian 16[1] and MOLPRO version 2012.1[2]. The geometrical parameters of all monomers and complexes were optimized by means of the double-hybrid density functional B2PLYP[3]. In this approximation a part of the correlation energy is calculated with second-order perturbation theory.[3] To consider the dispersion interaction in an appropriate way, the additional dispersion correction with Becke-Johnson damping[4] (D3BJ) was employed. As basis set TZVP (triple zeta valence basis set with polarization functions) was employed for the light elements C, H, N, P, O, S, Se and Cl, whereas aug-cc-pVTZ-PP (augmented correlation-consistent polarized valence triple zeta basis set) was applied for tellurium. For all structures C1 symmetry was applied. Frequency calculations were carried out at each of the stationary points to verify the nature of the stationary point. It turned out that all stationary states have none imaginary frequency. Furthermore, single point calculations on the B2PLYP-optimized structures were performed using B3LYP[5-7], B3LYP-D3[4], B2PLYP and CCSD(T)[8].

To calculated the relevant parts of the interaction energies in the complexes, the DFT-SAPT[9-12] (density-functional theory symmetry-adapted intermolecular perturbation theory) approximation was applied. In this approach, the total interaction energy E^{SAPT} between closed-shell molecules can be obtained as a sum of individual first and second-order interaction terms, namely, electrostatic $E^{(1)}_{ELST}$, induction $E^{(2)}_{ind}$, and dispersion $E^{(2)}_{disp}$ interaction. Each of these terms is accompanied by its exchange counterpart, abbreviated as $E^{(1)}_{EXCH}$, $E^{(2)}_{exch-ind}$ and $E^{(2)}_{exch-disp}$, respectively. The latter terms arise due to electron exchange between the monomers when the molecules are close to each other. Effects of higher than second order in the intermolecular potential can be estimated from the difference $\delta(HF)$ of counterpoise-corrected supermolecular HF calculations and electrostatic and induction energies along with their exchange counterparts obtained from static and response HF-level properties. In sum, using the SAPT treatment the total interaction energy E^{SAPT} between closed-shell molecules can be written as:

$$E^{SAPT} = E^{(1)}_{ELST} + E^{(1)}_{EXCH} + E^{(2)}_{IND} + E^{(2)}_{DISP} + \delta(HF)$$

with

$E^{(2)}_{IND} = E^{(2)}_{ind} + E^{(2)}_{exch-ind}$ and $E^{(2)}_{DISP} = E^{(2)}_{disp} + E^{(2)}_{exch-disp}$.

In order to achieve high accuracy, the wrong asymptotic behavior of the exchange-correlation potential was corrected by suppling a shift parameter, which should approximate the difference between the HOMO (highest occupied molecular orbital) energy obtained from the respective DFT (density-functional theory) calculation and the (negative) ionisation potential of the monomer. As DFT method PBE[13-14] was employed. Density fitting was used to approximate the integrals in SAPT.[10] The BSSE (basis set superposition error) was eliminated by introducing ghost orbitals, which are basis set functions having no electrons or protons. As basis set aug-cc-pVTZ (augmented correlation-consistent polarized valence triple zeta basis set) was employed for C and H, whereas aug-cc-pVQZ (augmented correlation-consistent polarized valence quadruple zeta basis set) was applied for the light elements N, P, O, S, Se and Cl. As fitting basis sets aug-cc-pVTZ/JKFIT, aug-cc-pVQZ/JKFIT, aug-cc-pVTZ/MP2FIT and aug-cc-pVQZ/MP2FIT were used. In the case of tellurium aug-cc-pVQZ-PP together with QZVPP/JKFIT and QZVPP/MP2FIT were applied. The latter are universal fitting basis sets and work well with the cc-pVXZ-PP and aug-cc-pVXZ-PP series of basis sets.[2]

3. Cartesian Coordinates and Absolute Energies for All Calculated Compounds

Table S12. Absolute energies [au] calculated by means of different methods.

Compound	B2PLYP-D3[a]	B2PLYP[b]	B3LYP-D3BJ[c]
1,3-thiazole	-568.8090013	-568.8027041	-569.1409270
1,3-selenazole	-2571.9025090	-2571.8956314	-2572.5069210
1,3-tellurazole	-438.5066396	-438.4987822	-439.0261220
1,3-thiazole N-oxide	-643.9310121	-643.9237003	-644.3220420
1,3-selenazole N-oxide	-2647.0253273	-2647.0174279	-2647.6885890
1,3-tellurazole N-oxide	-513.6321211	-513.6232284	-514.2102810
1,3,5-thiadiazole	-584.8424832	-584.8371633	-585.1750150
1,3,5-selenadiazole	-2587.9386502	-2587.9328097	-2588.5435680
1,3,5-telluradiazole	-454.5542745	-454.5475843	-455.0740480
1,2-thiazole N-oxide	-643.9289895	-643.9216925	-644.3200170
1,2-selenazole N-oxide	-2647.0301370	-2647.0222552	-2647.6935620
1,2-tellurazole N-oxide	-513.6451704	-513.6362858	-514.2235690
thiophene	-552.7649293	-552.7577658	-553.0953270
selenaphene	-2555.8585904	-2555.8508305	-2556.4612180
telluraphene	-422.4632560	-422.4544862	-422.9805820
acetamide	-209.1005766	-209.0967880	-209.3092140
pyridine	-248.1162583	-248.1084640	-248.3847920
trimethylamine	-174.3383859	-174.3322485	-174.5516920
trimethylphosphine	-460.8680397	-460.8598818	-461.1852990
tetrachloromethane	-1878.3048178	-1878.2974578	-1878.9849110
AcOH	-228.9750707	-228.9719952	-229.1856400
AcOH-Dimer	-457.9780552	-457.9699364	-458.4013060

[a] B2PLYP-D3/TZVP,aug-cc-pVTZ-PP
[b] B2PLYP/TZVP,aug-cc-pVTZ//B2PLYP-D3/TZVP,aug-cc-pVTZ-PP
[c] B3LYP-D3BJ/TZVP,aug-cc-pVTZ//B2PLYP-D3/TZVP,aug-cc-pVTZ-PP

Table S13. Absolute energies [au] calculated by means of different methods.

Compound	B3LYP[d]	CCSD(T)[e]
1,3-thiazole	-569.1273950	-568.1401869
1,3-selenazole	-2572.4925050	-2570.5204850
1,3-tellurazole	-439.0101560	-437.6592603
1,3-thiazole N-oxide	-644.3063060	-643.1503367
1,3-selenazole N-oxide	-2647.6719580	-2645.5319491
1,3-tellurazole N-oxide	-514.1920730	-512.6735632
1,3,5-thiadiazole	-585.1635480	-584.1565028
1,3,5-selenadiazole	-2588.5313000	-2586.5393627
1,3,5-telluradiazole	-455.0604410	-453.6896640
1,2-thiazole N-oxide	-644.3043390	-643.1459267
1,2-selenazole N-oxide	-2647.6770100	-2645.5328496
1,2-tellurazole N-oxide	-514.2054400	-512.6825722
thiophene	-553.0799790	-552.1122512
selenaphene	-2556.4449730	-2554.4932125
telluraphene	-422.9627540	-421.6332463
acetamide	-209.3005800	-208.7677026
pyridine	-248.3676210	-247.6885016
trimethylamine	-174.5377080	-174.0502151
trimethylphosphine	-461.1675920	-460.3005369
tetrachloromethane	-1878.9701180	-1876.6299438
AcOH	-229.178624	-228.6231598
AcOH-Dimer	-458.383129	-457.2709917

[d] B3LYP/TZVP,aug-cc-pVTZ//B2PLYP-D3/TZVP,aug-cc-pVTZ-PP
[e] CCSD(T)/TZVP,aug-cc-pVTZ//B2PLYP-D3/TZVP,aug-cc-pVTZ-PP

Table S14. Absolute energies [au] calculated by means of different methods.

	B2PLYP-D3[a]	B2PLYP[b]	B3LYP-D3BJ[c]	B3LYP[d]	CCSD(T)[e]
**1a**	-777.9176587	-777.9053802	-778.4588550	-778.4323480	-776.9154451
**1b**	-2781.0122223	-2780.9989450	-2781.8260900	-2781.7979030	-2779.2966031
**1c**	-647.6192180	-647.6042466	-648.3477850	-648.3167110	-646.4391343
**2a**	-777.9253407	-777.9131784	-778.4667990	-778.4404130	-776.9223770
**2b**	-2781.0187131	-2781.0059297	-2781.8326600	-2781.8053100	-2779.3025631
**2c**	-647.6225650	-647.6087564	-648.3515760	-648.3225920	-646.4410726
**3a**	-743.1555268	-743.1396891	-743.7013360	-743.6671270	-742.1982057
**3b**	-2746.2505593	-2746.2334680	-2747.0692250	-2747.0328460	-2744.5795841
**3c**	-612.8588687	-612.8396694	-613.5920320	-613.5520250	-611.7240342
**4a**	-1029.6830420	-1029.6654046	-1030.3328830	-1030.2955810	-1028.4460255
**4b**	-3032.7774309	-3032.7588614	-3033.6999330	-3033.6611730	-3030.8268371
**4c**	-899.3846627	-899.3642957	-900.2216180	-900.1799640	-897.9699907
**5a**	-2447.1189819	-2447.1011512	-2448.1316040	-2448.0952990	-2444.7748318
**5b**	-4450.2126925	-4450.1940232	-4451.4978870	-4451.4602560	-4447.1551520
**5c**	-2316.8180758	-2316.7984323	-2318.0174380	-2317.9784150	-2314.2971005
**6a**	-800.8875510	-800.8702341	-801.4866850	-801.4495540	-799.8069362
**6b**	-2803.9821181	-2803.9637463	-2804.8535700	-2804.8146910	-2802.1886043
**6c**	-670.5898900	-670.5697691	-671.3757970	-671.3340240	-669.3323260
**7a**	-816.9327619	-816.9162069	-817.5334420	-817.4979210	-815.8358851
**7b**	-2820.0275921	-2820.0100770	-2820.9009770	-2820.8638840	-2818.2171294
**7c**	-686.6349612	-686.6156659	-687.4231090	-687.3830190	-685.3598666
**8a**	-892.0568706	-892.0391610	-892.7166640	-892.6786590	-890.8480897
**8b**	-2895.1527799	-2895.1340963	-2896.0851030	-2896.0455120	-2893.2308314
**8c**	-761.7636092	-761.7430631	-762.6104780	-762.5677370	-760.3773308

[a] B2PLYP-D3/TZVP,aug-cc-pVTZ-PP
[b] B2PLYP/TZVP,aug-cc-pVTZ//B2PLYP-D3/TZVP,aug-cc-pVTZ-PP
[c] B3LYP-D3BJ/TZVP,aug-cc-pVTZ//B2PLYP-D3/TZVP,aug-cc-pVTZ-PP
[d] B3LYP/TZVP,aug-cc-pVTZ//B2PLYP-D3/TZVP,aug-cc-pVTZ-PP
[e] CCSD(T)/TZVP,aug-cc-pVTZ//B2PLYP-D3/TZVP,aug-cc-pVTZ-PP

Table S15. Absolute energies [au] calculated by means of different methods.

	B2PLYP-D3[a]	B2PLYP[b]	B3LYP-D3BJ[c]	B3LYP[d]	CCSD(T)[e]
9a	-832.9661977	-832.9506048	-833.5678020	-833.5342880	-831.8513854
9b	-2836.0662462	-2836.0494566	-2836.9407710	-2836.9051650	-2834.2371038
9c	-702.6894986	-702.6708276	-703.4783570	-703.4395340	-701.3959521
10a	-892.0556567	-892.0377223	-892.7156980	-892.6772770	-890.8442967
10b	-2895.1597857	-2895.1406431	-2896.0926540	-2896.0521920	-2893.2337198
10c	-761.7815522	-761.7605513	-762.6284760	-762.5849430	-760.3916543
11a	-1137.6249305	-1137.6075939	-1138.2888710	-1138.2526340	-1136.2874844
11b	-5143.8110090	-5143.7933540	-5145.0203560	-5144.9841040	-5141.0465239
11c	-877.0215182	-877.0005673	-878.0604670	-878.0189860	-875.3270795
12a	-1137.6249613	-1137.6100529	-1138.2889670	-1138.2573850	-1136.2870907
12b	-5143.8131601	-5143.7966739	-5145.0222710	-5144.9881550	-5141.0485815
12c	-877.0243070	-877.0049800	-878.0633960	-878.0245750	-875.3292957
13a	-1287.8815395	-1287.8639651	-1288.6641230	-1288.6267410	-1286.3206330
13b	-5294.0697375	-5294.0508237	-5295.3969500	-5295.3574700	-5291.0831435
13c	-1027.2828447	-1027.2607091	-1028.4390470	-1028.3942510	-1025.3664961
14a	-1169.6922312	-1169.6788962	-1170.3577850	-1170.3295890	-1168.3190321
14b	-5175.8901994	-5175.8746480	-5177.1011980	-5177.0692680	-5173.0887675
14c	-909.1369920	-909.1174014	-910.1778060	-910.1392740	-907.4036593
15a	-1287.8690702	-1287.8480446	-1288.6510180	-1288.6071640	-1286.3037092
15b	-5294.0725514	-5294.0529642	-5295.4003530	-5295.3598310	-5291.0765855
15c	-1027.3203145	-1027.2954429	-1028.4771420	-1028.4282340	-1025.3970732

[a] B2PLYP-D3/TZVP,aug-cc-pVTZ-PP
[b] B2PLYP/TZVP,aug-cc-pVTZ//B2PLYP-D3/TZVP,aug-cc-pVTZ-PP
[c] B3LYP-D3BJ/TZVP,aug-cc-pVTZ//B2PLYP-D3/TZVP,aug-cc-pVTZ-PP
[d] B3LYP/TZVP,aug-cc-pVTZ//B2PLYP-D3/TZVP,aug-cc-pVTZ-PP
[e] CCSD(T)/TZVP,aug-cc-pVTZ//B2PLYP-D3/TZVP,aug-cc-pVTZ-PP

Cartesian coordinates of the optimized geometry for 1,3-thiazole at B3LYP-D3BJ/TZVP,aug-cc-pVTZ-PP level of theory (number of imaginary frequencies = 0):

N	1.25883900	0.77929400	0.00000000
C	0.03182400	1.20598200	-0.00000100
C	1.28977500	-0.59559500	0.00000000
C	0.07859100	-1.21943900	0.00000100
H	-0.24867200	2.24744500	-0.00000100
H	2.24357100	-1.09883400	0.00000000
H	-0.13463000	-2.27421700	0.00000100
S	-1.19208000	-0.04219600	0.00000000

Cartesian coordinates of the optimized geometry for 1,3-selenazole at B3LYP-D3BJ/TZVP,aug-cc-pVTZ-PP level of theory (number of imaginary frequencies = 0):

N	1.68768100	-0.75006100	0.00002100
C	0.49725600	-1.25519500	0.00005800
C	1.68057400	0.62624700	0.00002500
C	0.47612300	1.25834500	0.00005400
H	0.29291700	-2.31507900	0.00006100
H	2.63008900	1.14023900	0.00002300
H	0.29141200	2.31916800	0.00007100
Se	-0.91035000	0.00969800	-0.00003300

Cartesian coordinates of the optimized geometry for 1,3-tellurazole at B3LYP-D3BJ/TZVP,aug-cc-pVTZ-PP level of theory (number of imaginary frequencies = 0):

Te	-0.77616400	0.00397400	-0.00006500
N	2.00779600	-0.73854000	-0.00010300
C	0.85447100	-1.31589700	0.00033800
C	1.98962700	0.63838800	-0.00009900
C	0.80931700	1.31518100	0.00033200
H	0.73486400	-2.39012400	0.00038700
H	2.94829700	1.13911900	-0.00024200
H	0.70233700	2.38812400	0.00050800

Cartesian coordinates of the optimized geometry for 1,3-thiazole *N*-oxide at B3LYP-D3BJ/TZVP,aug-cc-pVTZ-PP level of theory (number of imaginary frequencies = 0):

N	-1.04471100	-0.08631300	-0.00001500
C	-0.11787100	-1.05577200	-0.00096300
C	-0.46431400	1.19641300	-0.00019000
C	0.88255700	1.17644800	0.00013700
H	-0.39501300	-2.09358400	0.00098100
H	-1.13887700	2.03431100	0.00018700
H	1.55141400	2.01904700	-0.00090400
O	-2.30874400	-0.26103200	0.00029300
S	1.49769800	-0.44811600	0.00022400

Cartesian coordinates of the optimized geometry for 1,3-selenazole *N*-oxide at B3LYP-D3BJ/TZVP,aug-cc-pVTZ-PP level of theory (number of imaginary frequencies = 0):

N	-1.47336900	-0.15027400	-0.00032800
C	-0.45905800	-1.02297300	-0.00098400
C	-1.06370500	1.20219600	-0.00048900
C	0.26360400	1.40897100	0.00028200
H	-0.64924100	-2.08097700	0.00043600
H	-1.85864700	1.92832800	-0.00016200
H	0.76829400	2.35950800	-0.00029900
O	-2.71286600	-0.46075800	0.00066700
Se	1.21503100	-0.20582500	0.00012200

Cartesian coordinates of the optimized geometry for 1,3-tellurazole *N*-oxide at B3LYP-D3BJ/TZVP,aug-cc-pVTZ-PP level of theory (number of imaginary frequencies = 0):

N	-1.80906400	-0.19838600	-0.00000100
C	-0.78092400	-1.05374600	-0.00004900
C	-1.47971900	1.18186900	0.00000300
C	-0.18552000	1.53297800	-0.00001900
H	-0.98808200	-2.11027800	-0.00004300
H	-2.33765800	1.83527100	0.00000200
H	0.17934700	2.54672100	-0.00004500
O	-3.03971400	-0.54758300	0.00002400
Te	1.05393300	-0.12440300	0.00000600

Cartesian coordinates of the optimized geometry for 1,2-thiazole *N*-oxide at B3LYP-D3BJ/TZVP,aug-cc-pVTZ-PP level of theory (number of imaginary frequencies = 0):

C	-1.19517300	1.17410700	0.00003600
C	-1.60191300	-0.12615800	0.00035900
H	-1.87376200	2.01214300	-0.00001100
H	-2.60962700	-0.50674100	-0.00077400
N	0.89838300	0.18537100	-0.00006100
C	0.20393600	1.33301500	-0.00014800
H	0.77477600	2.24608700	-0.00018200
O	2.13265300	0.00966000	0.00012300
S	-0.25515000	-1.21325900	-0.00006700

Cartesian coordinates of the optimized geometry for 1,2-selenazole *N*-oxide at B3LYP-D3BJ/TZVP,aug-cc-pVTZ-PP level of theory (number of imaginary frequencies = 0):

C	0.16216900	1.89521100	0.00005800
C	-1.03096800	1.24302700	0.00032600
H	0.23601700	2.97255500	-0.00003800
H	-2.01428000	1.68337300	-0.00129400
N	1.13816100	-0.23442300	-0.00013800
C	1.32174300	1.09079800	-0.00006800
H	2.34498400	1.43045800	-0.00003300
O	1.99025700	-1.13979700	0.00006400
Se	-0.79922300	-0.60886100	-0.00000200

Cartesian coordinates of the optimized geometry for 1,2-tellurazole *N*-oxide at B3LYP-D3BJ/TZVP,aug-cc-pVTZ-PP level of theory (number of imaginary frequencies = 0):

Te	-0.85885800	-0.20180900	-0.00005700
C	1.22954100	1.72930500	0.00017200
C	-0.12994200	1.72731000	0.00081700
H	1.79026100	2.65500800	-0.00041500
H	-0.76367200	2.59925700	-0.00071600
N	1.24770900	-0.64253500	-0.00035300
C	1.93693400	0.50358900	-0.00057100
H	3.01227600	0.40500300	-0.00018100
O	1.70857500	-1.80358600	0.00053200

Cartesian coordinates of the optimized geometry for 1,3,5-thiadiazole at B3LYP-D3BJ/TZVP,aug-cc-pVTZ-PP level of theory (number of imaginary frequencies = 0):

S	1.11761500	-0.00125600	-0.00002000
C	-1.17539000	0.71178400	0.00000000
H	-2.04947500	1.34572200	-0.00005900
N	0.02223100	-1.25839300	0.00004100
C	-1.17796600	-0.70876700	-0.00001900
H	-2.05427500	-1.33971200	-0.00003900
N	0.02663200	1.25781900	0.00003500

Cartesian coordinates of the optimized geometry for 1,3,5-selenadiazole at B3LYP-D3BJ/TZVP,aug-cc-pVTZ-PP level of theory (number of imaginary frequencies = 0):

Se	-0.85827500	-0.00000100	0.00001800
C	1.56091100	0.71646900	-0.00039700
H	2.46057800	1.31856800	-0.00016000
N	0.39501400	-1.31779300	-0.00028400
C	1.56090500	-0.71634500	0.00029700
H	2.46067800	-1.31830700	0.00086000
N	0.39486900	1.31765300	0.00018500

Cartesian coordinates of the optimized geometry for 1,3,5-telluradiazole at B3LYP-D3BJ/TZVP,aug-cc-pVTZ-PP level of theory (number of imaginary frequencies = 0):

Te	0.73335400	-0.00000300	-0.00006200
C	-1.85920900	0.72332100	-0.00027400
H	-2.78813000	1.28683500	-0.00049300
N	-0.73173400	-1.38281700	0.00044400
C	-1.85920400	-0.72393000	-0.00015500
H	-2.78811400	-1.28727400	-0.00039000
N	-0.73222300	1.38342500	0.00051100

Cartesian coordinates of the optimized geometry for thiophene at B3LYP-D3BJ/TZVP,aug-cc-pVTZ-PP level of theory (number of imaginary frequencies = 0):

C	0.01285500	-1.23970100	0.00000000
C	1.27170300	0.71191800	0.00000000
C	0.01292400	1.23964800	0.00000000
H	-0.27800400	-2.27658900	0.00000100
H	2.16694300	1.31534100	-0.00000100
H	-0.27808600	2.27647700	-0.00000100
S	-1.19957400	0.00002100	0.00000000
C	1.27173500	-0.71193600	0.00000000
H	2.16702600	-1.31514300	0.00000000

Cartesian coordinates of the optimized geometry for selenaphene at B3LYP-D3BJ/TZVP,aug-cc-pVTZ-PP level of theory (number of imaginary frequencies = 0):

C	0.44377400	-1.28782200	-0.00001200
C	1.67760200	0.71410300	-0.00000800
C	0.44314200	1.28787600	-0.00000600
H	0.20383600	-2.33798100	-0.00001800
H	2.58760300	1.29740100	-0.00000700
H	0.20424500	2.33835500	-0.00000400
Se	-0.91293300	-0.00008300	0.00000800
C	1.67805300	-0.71389200	-0.00001200
H	2.58859700	-1.29653800	-0.00002100

Cartesian coordinates of the optimized geometry for telluraphene at B3LYP-D3BJ/TZVP,aug-cc-pVTZ-PP level of theory (number of imaginary frequencies = 0):

Te	-0.77635400	0.00001200	0.00005300
C	0.78530600	-1.35218100	0.00002300
C	1.98791600	0.71599200	-0.00024700
C	0.78521100	1.35211000	0.00010600
H	0.62828200	-2.41915700	0.00010300
H	2.91722400	1.27237900	-0.00039300
H	0.62921300	2.41925800	0.00025200
C	1.98795300	-0.71605900	-0.00026200
H	2.91735400	-1.27225100	-0.00043000

Cartesian coordinates of the optimized geometry for acetamid at B3LYP-D3BJ/TZVP,aug-cc-pVTZ-PP level of theory (number of imaginary frequencies = 0):

O	0.36031700	1.33166000	0.00258000
C	0.07522700	0.14627000	-0.00312100
N	1.02866300	-0.83127600	-0.01640200
C	-1.35936700	-0.34095600	-0.00000100
H	-1.45244100	-1.41820500	-0.12795800
H	-1.82093400	-0.05344000	0.94383100
H	-1.89752200	0.16403500	-0.79927200
H	1.99367300	-0.55357900	0.04293500
H	0.79888800	-1.80504000	0.05337600

Cartesian coordinates of the optimized geometry for pyridine at B3LYP-D3BJ/TZVP,aug-cc-pVTZ-PP level of theory (number of imaginary frequencies = 0):

C	-1.14031500	-0.71939500	-0.00002300
C	-1.19468200	0.67102500	-0.00018300
C	-0.00007000	1.38127900	0.00064800
C	1.19462100	0.67112400	-0.00026100
C	1.14039100	-0.71928000	-0.00009600
H	-2.05317100	-1.30317900	-0.00029500
H	-2.14958500	1.17798100	0.00052200
H	-0.00010200	2.46312100	0.00007700
H	2.14946300	1.17819600	0.00037900
H	2.05328800	-1.30299600	-0.00043000
N	0.00006300	-1.41737700	-0.00010900

Cartesian coordinates of the optimized geometry for trimethylamine at B3LYP-D3BJ/TZVP,aug-cc-pVTZ-PP level of theory (number of imaginary frequencies = 0):

N	0.00000000	0.00000400	-0.38152200
C	-0.92198300	-1.03033600	0.06209400
H	-0.98372700	-1.09932200	1.16222900
H	-1.92033200	-0.82212700	-0.32227500
H	-0.60462300	-1.99950900	-0.32230300
C	-0.43131000	1.31362300	0.06209700
H	-0.46037500	1.40150900	1.16222800
H	0.24825300	2.07410600	-0.32213200
H	-1.42926200	1.52340600	-0.32244600
C	1.35329900	-0.28328900	0.06209300
H	1.67213900	-1.25200500	-0.32222400
H	2.03393900	0.47612700	-0.32234800
H	1.44395000	-0.30219700	1.16222200

Cartesian coordinates of the optimized geometry for trimethylphosphine at B3LYP-D3BJ/TZVP,aug-cc-pVTZ-PP level of theory (number of imaginary frequencies = 0):

P	-0.00013800	-0.00020600	-0.61055000
C	1.22144300	-1.08370500	0.28289100
H	1.12544300	-1.00050100	1.36718900
H	1.06801800	-2.12300500	-0.00692300
H	2.23492300	-0.80458600	-0.00471700
C	-1.54941600	-0.51556500	0.28298100
H	-1.42897600	-0.47517300	1.36727900
H	-2.37185300	0.13852800	-0.00600300
H	-1.81614400	-1.53210100	-0.00549800
C	0.32812800	1.59950600	0.28274200
H	1.30597400	1.98460600	-0.00576400
H	-0.41866600	2.33883800	-0.00604800
H	0.30242500	1.47507200	1.36704300

Cartesian coordinates of the optimized geometry for tetrachloromethane at B3LYP-D3BJ/TZVP,aug-cc-pVTZ-PP level of theory (number of imaginary frequencies = 0):

Cl	-0.24729300	0.69716500	-1.62626400
C	-0.00048500	-0.00032900	-0.00026100
Cl	-0.31568800	1.24981800	1.23744500
Cl	1.68567100	-0.57632800	0.15135800
Cl	-1.12251900	-1.37054000	0.23755300

Cartesian coordinates of the optimized geometry for AcOH at B3LYP-D3BJ/TZVP,aug-cc-pVTZ-PP level of theory (number of imaginary frequencies = 0):

O	0.62998700	1.20577900	-0.00000600
C	0.08881800	0.12741700	0.00002700
C	-1.39110900	-0.12163900	0.00000300
H	-1.66680900	-0.70224300	-0.87931200
H	-1.66683500	-0.70252900	0.87911300
H	-1.91674500	0.82722500	0.00011600
H	1.72615000	-0.79815200	-0.00000900
O	0.78726200	-1.03815000	-0.00000500

Cartesian coordinates of the optimized geometry for AcOH-AcOH at B3LYP-D3BJ/TZVP,aug-cc-pVTZ-PP level of theory (number of imaginary frequencies = 0):

O	-1.22954300	1.06413700	-0.00080000
C	-1.92560300	0.05454000	-0.00039100
C	-3.42471500	0.09470000	0.00057100
H	-3.80308300	-0.42559900	-0.87843700
H	-3.80224400	-0.42741100	0.87885500
H	-3.76555100	1.12445500	0.00172100
H	-0.44129400	-1.14663700	0.00000300
O	-1.43851500	-1.17544100	0.00069300
H	0.44098600	1.14604300	-0.00022400
O	1.43826200	1.17531600	0.00023300
C	1.92566300	-0.05454700	-0.00018100
O	1.22974500	-1.06424600	-0.00137000
C	3.42475700	-0.09444300	0.00080500
H	3.80199100	0.42626500	0.88005400
H	3.76576100	-1.12414300	0.00039900
H	3.80323300	0.42739200	-0.87724100

Cartesian coordinates of the optimized geometry for **1a** at B3LYP-D3BJ/TZVP,aug-cc-pVTZ-PP level of theory (number of imaginary frequencies = 0):

N	2.34462900	1.34492500	0.48483800
C	1.22344200	0.96913200	-0.05087200
C	3.24676400	0.30527600	0.50466800
C	2.80804700	-0.87363800	-0.01721000
O	-1.79358500	0.60148200	-0.72571600
C	-2.58425300	0.02022500	0.00523300
N	-2.20309600	-1.00540500	0.81340900
H	0.35137200	1.58653300	-0.18901000
H	4.22751200	0.47036600	0.92302400
H	3.34209600	-1.80376100	-0.10938200
C	-4.04830500	0.39783000	0.06010200
H	-4.63885000	-0.24836900	0.70734600
H	-4.12828600	1.42533900	0.41191400
H	-4.45235800	0.36133000	-0.94968500
H	-1.23007300	-1.26478600	0.83254700
H	-2.83980300	-1.45187600	1.44685100
S	1.17826000	-0.69851400	-0.58544600

Cartesian coordinates of the optimized geometry for **1b** at B3LYP-D3BJ/TZVP,aug-cc-pVTZ-PP level of theory (number of imaginary frequencies = 0):

N	1.98860300	1.72615900	-0.07228500
C	0.90703000	1.10566800	-0.41210900
C	2.97054500	0.87427100	0.38673800
C	2.69112600	-0.45615600	0.42500400
O	-1.99623100	0.05212400	-0.89957500
C	-2.78034200	0.09925600	0.03987700
N	-2.37181700	-0.01978000	1.33103000
H	0.01701100	1.57098500	-0.80433500
H	3.91207100	1.30427700	0.69511100
H	3.34487200	-1.24771200	0.75112900
C	-4.26610300	0.28967200	-0.17085900
H	-4.83543700	0.31854300	0.75658600
H	-4.42248200	1.21819700	-0.71707900
H	-4.63382700	-0.52537800	-0.79184100
Se	0.95697600	-0.77561000	-0.19809100
H	-1.38964700	-0.14435800	1.51713700
H	-3.01091300	0.03825900	2.10184900

Cartesian coordinates of the optimized geometry for **1c** at B3LYP-D3BJ/TZVP,aug-cc-pVTZ-PP level of theory (number of imaginary frequencies = 0):

N	1.78988600	1.91404300	-0.46141100
C	0.69982200	1.24563700	-0.63374000
C	2.82995900	1.21795600	0.12151500
C	2.67259000	-0.08713800	0.47219700
O	-2.09394300	-0.30744300	-0.76261700
C	-2.89116700	0.21359700	0.01141500
N	-2.48895800	0.96709300	1.06656600
H	-0.18745100	1.65590500	-1.09528000
H	3.75119900	1.76391100	0.27591100
H	3.43748200	-0.69411800	0.93130100
C	-4.38385000	0.05234700	-0.16272000
H	-4.96677200	0.66937100	0.51882100
H	-4.64205600	0.30394200	-1.18909700
H	-4.63948900	-0.99455000	-0.00334300
H	-1.50229300	1.04642200	1.25798300
H	-3.14219000	1.34433600	1.72817400
Te	0.76870200	-0.74342300	0.01131500

Cartesian coordinates of the optimized geometry for **2a** at B3LYP-D3BJ/TZVP,aug-cc-pVTZ-PP level of theory (number of imaginary frequencies = 0):

N	-0.85828500	0.83450900	0.00005900
C	-0.96655300	-0.46588800	0.00023000
C	-2.09631400	1.43342700	-0.00017000
C	-3.16062300	0.58476700	-0.00020700
O	2.08698000	-1.15881400	-0.00025600
C	2.73656400	-0.11665500	0.00006900
N	2.15873200	1.10773200	0.00021900
H	-0.10896500	-1.12623100	0.00047900
H	-2.16218700	2.51007400	-0.00028700
H	-4.20988300	0.82387200	-0.00035800
C	4.25172600	-0.14097600	-0.00001600
H	4.70215300	0.85043000	-0.00001500
H	4.58971700	-0.68754300	0.87880900
H	4.58963700	-0.68751100	-0.87888900
H	1.14298400	1.18775100	0.00022700
H	2.71699400	1.94109400	0.00035800
S	-2.60301300	-1.05657300	0.00002100

Cartesian coordinates of the optimized geometry for **2b** at B3LYP-D3BJ/TZVP,aug-cc-pVTZ-PP level of theory (number of imaginary frequencies = 0):

N	0.23462200	1.06722900	-0.00001300
C	0.43590300	-0.21645800	-0.00003500
C	1.40014600	1.80039500	0.00001800
C	2.57536400	1.11786000	-0.00000100
O	-2.55279300	-1.16421700	-0.00009700
C	-3.28077100	-0.17529800	-0.00010300
N	-2.79905200	1.09005900	-0.00003700
H	-0.37561500	-0.93382500	-0.00006300
H	1.32770900	2.87780300	0.00001600
H	3.57118200	1.52722400	-0.00000700
C	-4.78950100	-0.31635800	-0.00014100
H	-5.31506400	0.63740900	-0.00001300
H	-5.08433900	-0.88724400	-0.87910400
H	-5.08436800	-0.88750000	0.87864600
H	-1.79264900	1.24892500	-0.00001700
H	-3.41986400	1.87799300	-0.00003900
Se	2.24998600	-0.72085000	0.00009600

Cartesian coordinates of the optimized geometry for **2c** at B3LYP-D3BJ/TZVP,aug-cc-pVTZ-PP level of theory (number of imaginary frequencies = 0):

N	0.27004400	1.17375300	0.00001600
C	0.01112200	-0.09599900	0.00003100
C	-0.81858000	2.01949800	-0.00001300
C	-2.08023300	1.51430500	-0.00002000
O	2.96018100	-1.19502400	0.00006000
C	3.73109300	-0.23930300	0.00003400
N	3.30576500	1.04627600	0.00000700
H	0.80149800	-0.83854700	0.00006000
H	-0.61461100	3.08188700	-0.00001100
H	-2.98730500	2.09702600	-0.00002800
C	5.23218200	-0.44666400	0.00014000
H	5.79930800	0.48299100	0.00002500
H	5.50153300	-1.02996400	0.87913100
H	5.50160700	-1.03023600	-0.87864500
H	2.30741400	1.24986800	0.00000700
H	3.96079700	1.80601400	0.00004100
Te	-2.02761300	-0.54442500	-0.00004300

Cartesian coordinates of the optimized geometry for **3a** at B3LYP-D3BJ/TZVP,aug-cc-pVTZ-PP level of theory (number of imaginary frequencies = 0):

N	-2.34399300	-1.37978600	-0.00644400
C	-1.12605400	-0.92633700	-0.01537600
C	-3.24425300	-0.33969600	0.00474700
C	-2.70481700	0.91185800	0.00422100
H	-0.23837100	-1.53512900	-0.02532000
H	-4.30036900	-0.56040500	0.01310500
H	-3.21658300	1.85902700	0.01168700
S	-0.97355600	0.81242200	-0.01096600
N	2.03652800	-0.06243000	-0.00264800
C	2.60599400	-1.39561300	-0.11742600
H	3.70826200	-1.38702900	-0.09864300
H	2.25856900	-2.01817400	0.70745500
H	2.28596500	-1.85454800	-1.05306700
C	2.39375600	0.55110100	1.26667500
H	3.48242100	0.68430200	1.37788700
H	1.91777600	1.52770300	1.34895100
H	2.03773000	-0.07016600	2.08821000

C	2.43917200	0.77505100	-1.12138100
H	2.11680600	0.31842900	-2.05685200
H	1.96338300	1.75172300	-1.03877800
H	3.53077600	0.92284500	-1.16430200

Cartesian coordinates of the optimized geometry for **3b** at B3LYP-D3BJ/TZVP,aug-cc-pVTZ-PP level of theory (number of imaginary frequencies = 0):

N	2.15356000	1.60901900	0.00032100
C	0.93558800	1.17353900	0.00111800
C	3.07975400	0.58800700	-0.00070500
C	2.61413000	-0.69005900	-0.00069700
H	0.06451400	1.80817900	0.00210400
H	4.12666700	0.85359700	-0.00145600
H	3.20195800	-1.59293200	-0.00143500
Se	0.74213700	-0.70337300	0.00071700
N	-2.19168300	0.13137100	0.00006100
C	-2.59354300	0.83920400	-1.20588200
H	-3.68803500	0.93422900	-1.29217800
H	-2.16438700	1.84106200	-1.20838200
H	-2.22314300	0.30882600	-2.08260700
C	-2.59775800	0.85113600	1.19748900
H	-3.69254500	0.94695500	1.27903600
H	-2.23040000	0.32956500	2.08075500
H	-2.16869900	1.85302700	1.19147400
C	-2.71453200	-1.22624500	0.00589200
H	-2.35728100	-1.76114900	-0.87373000
H	-2.36031400	-1.75240600	0.89198900
H	-3.81596200	-1.25050300	0.00409500

Cartesian coordinates of the optimized geometry for **3c** at B3LYP-D3BJ/TZVP,aug-cc-pVTZ-PP level of theory (number of imaginary frequencies = 0):

N	2.11377500	1.78721500	-0.00010700
C	0.87717600	1.42099800	-0.00049900
C	3.03913900	0.75987000	0.00044800
C	2.62579200	-0.53482400	0.00044800
H	0.06152200	2.13012600	-0.00115800
H	4.08124400	1.05100200	0.00083900
H	3.28884200	-1.38648200	0.00088000
Te	0.55663400	-0.64395300	-0.00025000
N	-2.27947700	0.21731500	0.00003300
C	-2.61763100	0.96766500	-1.20313100
H	-3.69842900	1.16179000	-1.28071600
H	-2.09637400	1.92406300	-1.20131800
H	-2.30093200	0.40859500	-2.08281100
C	-2.61613900	0.96647400	1.20436200
H	-3.69706000	1.15891800	1.28434400
H	-2.29671000	0.40737800	2.08304900
H	-2.09646700	1.92376400	1.20209400
C	-2.93204500	-1.08552300	-0.00015000
H	-2.62989200	-1.64667600	-0.88416800
H	-2.62945100	-1.64711400	0.88343900
H	-4.02910600	-0.99945800	0.00018000

Cartesian coordinates of the optimized geometry for **4a** at B3LYP-D3BJ/TZVP,aug-cc-pVTZ-PP level of theory (number of imaginary frequencies = 0):

N	-2.74452400	-1.39078600	-0.21798400
C	-1.61059300	-0.85893500	-0.56221700
C	-3.59751100	-0.43163200	0.27632500
C	-3.10853500	0.83964300	0.30954600
H	-0.77280700	-1.39698400	-0.97505000

H	-4.58222000	-0.72448100	0.60585300
H	-3.59644600	1.73669200	0.65003900
S	-1.48981000	0.86767000	-0.31099300
P	2.00019500	-0.12073100	-0.26997700
C	3.72842100	-0.70101900	-0.63515800
H	4.41530700	-0.47585100	0.18268700
H	3.72292000	-1.77714300	-0.80653900
H	4.08994700	-0.22037300	-1.54383800
C	1.77406100	-0.87249300	1.41513900
H	2.60418100	-0.63399800	2.08254100
H	0.84739500	-0.50458200	1.85459400
H	1.69206100	-1.95555800	1.32522600
C	2.36848900	1.63112600	0.23056500
H	2.68299800	2.20208600	-0.64273800
H	1.46398400	2.09297100	0.62497500
H	3.15237900	1.68082900	0.98847500

Cartesian coordinates of the optimized geometry for **4b** at B3LYP-D3BJ/TZVP,aug-cc-pVTZ-PP level of theory (number of imaginary frequencies = 0):

N	-2.46955800	1.66945500	0.16985100
C	-1.32323100	1.13531800	0.43706800
C	-3.40083600	0.74679500	-0.25321800
C	-3.01526900	-0.55523200	-0.33312500
H	-0.45793000	1.67634300	0.78609400
H	-4.39053200	1.10040900	-0.50197100
H	-3.61510600	-1.39489900	-0.64179300
Se	-1.22820200	-0.73791500	0.18417200
P	2.23989900	0.14026200	0.21846000
C	3.91213900	0.59203300	0.89267500
H	4.69717100	0.47909800	0.14283000
H	3.89873000	1.62519900	1.23879200
H	4.14398700	-0.04322800	1.74707900
C	2.24396900	1.15940000	-1.33548700
H	3.14426400	0.99322400	-1.92976000
H	1.36971800	0.90785000	-1.93528400
H	2.17895200	2.21699600	-1.08020700
C	2.64080200	-1.52228200	-0.50845500
H	2.82311100	-2.23746400	0.29332300
H	1.79024900	-1.87897800	-1.08861100
H	3.51921400	-1.48174300	-1.15496900

Cartesian coordinates of the optimized geometry for **4c** at B3LYP-D3BJ/TZVP,aug-cc-pVTZ-PP level of theory (number of imaginary frequencies = 0):

N	-2.29629600	1.89825900	0.09841400
C	-1.11956400	1.40502800	0.27696300
C	-3.29295800	0.98245500	-0.17714700
C	-3.00669000	-0.34497900	-0.24508800
H	-0.25007400	2.00565300	0.50447100
H	-4.28710600	1.38086100	-0.33098400
H	-3.72818100	-1.11932900	-0.45521200
Te	-0.99572000	-0.67849800	0.10913900
P	2.34519300	0.15412100	0.14344200
C	3.83318600	0.50074600	1.19943000
H	4.75121500	0.52432900	0.60961000
H	3.70811900	1.45993800	1.70078000
H	3.92241100	-0.26894300	1.96537200
C	2.58458600	1.42602600	-1.18748300
H	3.58279900	1.37246100	-1.62563900
H	1.84238000	1.27216600	-1.97029800
H	2.43293700	2.42294900	-0.77402100
C	2.93189000	-1.36001500	-0.75553900
H	2.98731900	-2.19866100	-0.06179400

H	2.21610400	-1.61666800	-1.53612300
H	3.91298600	-1.20804600	-1.20873200

Cartesian coordinates of the optimized geometry for **5a** at B3LYP-D3BJ/TZVP,aug-cc-pVTZ-PP level of theory (number of imaginary frequencies = 0):

N	-3.05041400	-1.09219800	0.83495700
C	-2.85457700	0.15156700	1.15794400
C	-2.86625700	-1.27189200	-0.51543200
C	-2.52655000	-0.15927100	-1.22380500
H	-2.94118100	0.53714400	2.16169000
H	-2.99392200	-2.25675300	-0.93650700
H	-2.33172700	-0.06705100	-2.27797400
S	-2.42576100	1.20663500	-0.16496000
Cl	1.46986100	1.61715400	0.70176500
C	1.59098100	-0.03249400	0.02140700
Cl	0.43708800	-1.10785200	0.85017800
Cl	3.25491100	-0.64676900	0.24668300
Cl	1.21286400	0.01972700	-1.72763900

Cartesian coordinates of the optimized geometry for **5b** at B3LYP-D3BJ/TZVP,aug-cc-pVTZ-PP level of theory (number of imaginary frequencies = 0):

N	2.54783500	1.64822300	0.74021300
C	2.52916600	0.45037300	1.22909800
C	2.36686700	1.66690100	-0.62392000
C	2.19632600	0.47773000	-1.26100100
H	2.65368600	0.22688600	2.27775700
H	2.36717000	2.62327600	-1.12514400
H	2.04013700	0.31332000	-2.31339700
Se	2.25885400	-0.92718200	-0.03662700
Cl	-1.71758500	-1.52318200	0.89286400
C	-1.94673200	0.02058000	0.01861800
Cl	-0.90126600	1.27392000	0.73242800
Cl	-3.65867500	0.52262700	0.14137700
Cl	-1.52074700	-0.20691600	-1.70503100

Cartesian coordinates of the optimized geometry for **5c** at B3LYP-D3BJ/TZVP,aug-cc-pVTZ-PP level of theory (number of imaginary frequencies = 0):

N	2.88591500	1.94321800	-0.10877200
C	1.77081900	1.37395400	-0.41521900
C	3.87678700	1.10975800	0.36264200
C	3.65410200	-0.22679100	0.48408500
H	0.91475000	1.91456300	-0.79250500
H	4.81926300	1.57174200	0.62410500
H	4.37136400	-0.94753600	0.84340000
Te	1.74207300	-0.70192600	-0.11379000
Cl	-1.53531900	0.08951600	-1.52552700
C	-2.39206100	0.09324600	0.05262000
Cl	-2.15276600	-1.48479800	0.85349100
Cl	-4.13195100	0.36964700	-0.22529900
Cl	-1.73011000	1.39374700	1.07961300

Cartesian coordinates of the optimized geometry for **6a** at B3LYP-D3BJ/TZVP,aug-cc-pVTZ-PP level of theory (number of imaginary frequencies = 0):

C	3.41563100	0.84843100	-0.51258500
C	2.98696900	-0.94664600	0.89750800
C	1.76497800	-0.81844300	0.30205200
H	3.90680800	1.65483200	-1.03075200
H	3.20472100	-1.69687500	1.64308300

H	0.85921200	-1.38407700	0.44693000
S	1.75330900	0.48085100	-0.84747600
C	3.93462600	0.00900900	0.43133700
H	4.95453700	0.06881300	0.78120300
N	-1.51427800	-0.78686800	0.09844800
C	-2.59456800	-1.16024300	-0.59592800
C	-1.60437400	0.34074100	0.81249000
C	-3.78675500	-0.44405100	-0.60751500
H	-2.49794800	-2.07539500	-1.16748000
C	-2.75118500	1.12568100	0.86755800
H	-0.71528600	0.62530000	1.36202100
C	-3.86535300	0.72533100	0.13976000
H	-4.62647200	-0.79572200	-1.19058100
H	-2.76320100	2.02837300	1.46226800
H	-4.77516800	1.31034700	0.15572200

Cartesian coordinates of the optimized geometry for **6b** at B3LYP-D3BJ/TZVP,aug-cc-pVTZ-PP level of theory (number of imaginary frequencies = 0):

C	3.24414200	0.43480900	0.54320500
C	2.70836800	-1.42027500	-0.79632200
C	1.45962900	-0.87765800	-0.76551900
H	3.81393200	1.12819200	1.14004500
H	2.92966400	-2.33276800	-1.33271800
H	0.55688800	-1.22942200	-1.23354500
Se	1.41402100	0.69625100	0.24176700
C	3.69984200	-0.69393600	-0.06944200
H	4.73106800	-1.01377000	-0.00947800
N	-1.65881900	0.04972400	-0.35785600
C	-2.02014800	-0.71132500	0.68115500
C	-2.63377800	0.64794900	-1.05047200
C	-3.34227700	-0.90701300	1.06547100
H	-1.21004100	-1.17982900	1.22723500
C	-3.98427400	0.51667800	-0.74459000
H	-2.31510700	1.25993800	-1.88559200
C	-4.34535900	-0.27901900	0.33640600
H	-3.57409900	-1.53420100	1.91493900
H	-4.72869700	1.02574400	-1.34077000
H	-5.38544700	-0.40574900	0.60544000

Cartesian coordinates of the optimized geometry for **6c** at B3LYP-D3BJ/TZVP,aug-cc-pVTZ-PP level of theory (number of imaginary frequencies = 0):

Te	1.13256400	-0.63353000	-0.06202300
C	1.26737300	1.41453300	0.15407800
C	3.58941600	0.84056200	0.07673700
C	3.20025300	-0.45691400	-0.05756100
H	0.38408500	2.02768400	0.22701400
H	4.63566700	1.12231700	0.09894400
H	3.86882500	-1.29931600	-0.15207200
C	2.56047400	1.83315700	0.18933500
H	2.80998500	2.88200000	0.29916000
N	-1.78699300	0.16804400	0.03118500
C	-2.49023600	-0.01771600	1.15353100
C	-2.46606600	0.33518600	-1.10888400
C	-3.88009700	-0.03708100	1.18751100
H	-1.91000100	-0.15419600	2.05815100
C	-3.85483700	0.33182700	-1.17612000
H	-1.86660900	0.47853500	-1.99981200
C	-4.57574300	0.13968500	-0.00307100
H	-4.39883100	-0.19088700	2.12335900
H	-4.35339900	0.47210300	-2.12496800
H	-5.65731100	0.12955300	-0.01620100

Cartesian coordinates of the optimized geometry for **7a** at B3LYP-D3BJ/TZVP,aug-cc-pVTZ-PP level of theory (number of imaginary frequencies = 0):

N	-3.02430500	-0.49214700	1.26075800
C	-1.82907500	-0.52031600	0.75065400
C	-3.91244500	0.07507900	0.37679900
C	-3.38779300	0.48176000	-0.81347900
H	-0.94956900	-0.91135700	1.23429500
H	-4.94910100	0.16786500	0.66140100
H	-3.89437800	0.94494100	-1.64283800
S	-1.68741900	0.14714100	-0.85865800
N	1.32811200	-0.38524400	0.10866700
C	1.69551000	0.86077600	0.42784600
C	2.29422100	-1.23639300	-0.25324000
C	3.01377300	1.30265400	0.40383300
H	0.89450400	1.53201500	0.71376500
C	3.64014700	-0.89078400	-0.31083400
H	1.97233400	-2.23883200	-0.50806500
C	4.00694100	0.40711000	0.02502900
H	3.25004900	2.32257100	0.67318000
H	4.37669700	-1.62259600	-0.61180900
H	5.04385600	0.71356500	-0.00702400

Cartesian coordinates of the optimized geometry for **7b** at B3LYP-D3BJ/TZVP,aug-cc-pVTZ-PP level of theory (number of imaginary frequencies = 0):

N	2.65850500	1.55850200	0.59724900
C	1.47298800	1.04900600	0.50692000
C	3.63932100	0.70282200	0.14486400
C	3.25307200	-0.51656000	-0.31898500
H	0.56298500	1.54402900	0.80510600
H	4.66437500	1.04150700	0.18034400
H	3.89081600	-1.29443700	-0.70468300
Se	1.39516700	-0.69920200	-0.20679700
N	-1.58203200	0.06833100	0.18542200
C	-2.40789300	-0.37801700	1.13807600
C	-2.13393300	0.54406300	-0.93633800
C	-3.79315700	-0.36900100	1.01657600
H	-1.93495600	-0.75956900	2.03486800
C	-3.50655700	0.59643700	-1.15224100
H	-1.44168500	0.89562100	-1.69208100
C	-4.35346800	0.13024800	-0.15345900
H	-4.41165800	-0.74293500	1.82043500
H	-3.89620200	0.99282800	-2.07937700
H	-5.42691700	0.15401300	-0.28467500

Cartesian coordinates of the optimized geometry for **7c** at B3LYP-D3BJ/TZVP,aug-cc-pVTZ-PP level of theory (number of imaginary frequencies = 0):

N	2.40835300	1.93069600	-0.00046700
C	1.22350500	1.42250200	-0.00035000
C	3.44763500	1.01955600	-0.00024500
C	3.19070300	-0.31575800	0.00006800
H	0.31952800	2.01495000	-0.00048900
H	4.44890900	1.42984500	-0.00034300
H	3.94932000	-1.08331000	0.00024800
Te	1.15127300	-0.66673600	0.00016200
N	-1.74810700	0.09376000	-0.00001300
C	-2.43856400	0.11712100	1.14550500
C	-2.43855800	0.11627900	-1.14555000
C	-3.82684900	0.16875300	1.19626500
H	-1.84930500	0.09523200	2.05426800
C	-3.82684300	0.16788200	-1.19635400

H	-1.84929800	0.09370700	-2.05429600
C	-4.53453700	0.19403800	-0.00005600
H	-4.33528500	0.18711200	2.14996500
H	-4.33527500	0.18554700	-2.15007000
H	-5.61544900	0.23371900	-0.00007200

Cartesian coordinates of the optimized geometry for **8a** at B3LYP-D3BJ/TZVP,aug-cc-pVTZ-PP level of theory (number of imaginary frequencies = 0):

N	-3.52903200	-0.07722300	0.13664000
C	-2.87505500	-0.26925200	-1.01799100
C	-2.66101000	0.32937800	1.16737200
C	-1.37537500	0.43654000	0.77783500
H	-3.39724600	-0.58008300	-1.90409000
H	-3.10279700	0.50620100	2.13214000
H	-0.51130000	0.72236900	1.34926400
O	-4.78442000	-0.23296600	0.32339800
S	-1.17817700	0.03577600	-0.89939800
N	1.74073600	0.33402600	0.15235900
C	2.11908200	-0.93846500	0.31922100
C	2.70134900	1.23590100	-0.07880300
C	3.44355700	-1.35810100	0.26811500
H	1.32215900	-1.65014400	0.49955100
C	4.05265500	0.91552100	-0.15068200
H	2.37029600	2.25851700	-0.21281900
C	4.43097800	-0.41014300	0.02648700
H	3.68905000	-2.40111300	0.41081700
H	4.78452900	1.68774200	-0.34180300
H	5.47248900	-0.69807200	-0.02219500

Cartesian coordinates of the optimized geometry for **8b** at B3LYP-D3BJ/TZVP,aug-cc-pVTZ-PP level of theory (number of imaginary frequencies = 0):

N	3.38657200	-0.39554500	-0.00148700
C	2.83528200	0.82304400	0.00152200
C	2.44080000	-1.44461800	-0.00339500
C	1.15129500	-1.06701100	-0.00179700
H	3.46593700	1.69427800	0.00333000
H	2.85142600	-2.44005100	-0.00582100
H	0.27796800	-1.69368400	-0.00286100
O	4.64299700	-0.64436400	-0.00266200
Se	0.96645400	0.79472500	0.00252700
N	-1.89466800	-0.10770300	0.00067500
C	-2.58857400	-0.13425400	1.14418100
C	-2.58669900	-0.12679100	-1.14411900
C	-3.97712900	-0.18199200	1.19460500
H	-2.00239500	-0.11341300	2.05501400
C	-3.97516900	-0.17417900	-1.19713100
H	-1.99901900	-0.10002700	-2.05383000
C	-4.68434600	-0.20408000	-0.00192800
H	-4.48578800	-0.20210100	2.14814200
H	-4.48226200	-0.18804800	-2.15161200
H	-5.76535200	-0.24064900	-0.00293400

Cartesian coordinates of the optimized geometry for **8c** at B3LYP-D3BJ/TZVP,aug-cc-pVTZ-PP level of theory (number of imaginary frequencies = 0):

N	3.28105500	0.64808700	-0.00130600
C	2.84290900	-0.61340600	0.00073700
C	2.27998000	1.65523000	-0.00268800
C	0.99280600	1.27951200	-0.00188500
H	3.58267200	-1.39732900	0.00181000
H	2.67348700	2.65945200	-0.00435000

H	0.14555000	1.94437600	-0.00283000
O	4.51476000	1.00078200	-0.00207800
Te	0.77541500	-0.77456200	0.00143900
N	-1.99477900	0.08006000	0.00019100
C	-2.67901200	0.15915900	-1.14710700
C	-2.67928400	0.16604800	1.14683100
C	-4.05714600	0.33177700	-1.19746200
H	-2.09318500	0.08155200	-2.05490100
C	-4.05742700	0.33897400	1.19582000
H	-2.09368200	0.09387200	2.05521700
C	-4.75957700	0.42290300	-0.00116700
H	-4.56227700	0.39069200	-2.15116600
H	-4.56278400	0.40361700	2.14903400
H	-5.83285100	0.55650600	-0.00169300

Cartesian coordinates of the optimized geometry for **9a** at B3LYP-D3BJ/TZVP,aug-cc-pVTZ-PP level of theory (number of imaginary frequencies = 0):

S	-1.67653300	-0.79377500	0.00086700
C	-3.85597700	0.24498000	-0.00090000
H	-4.92844100	0.37346000	-0.00143100
N	-1.63794200	0.87871100	-0.00042500
C	-2.89340200	1.28739600	-0.00115400
H	-3.10832300	2.34592000	-0.00203800
N	-3.33624500	-0.96784700	0.00024000
N	1.26059100	-0.33811700	0.00090800
C	1.62656900	0.94858400	0.00142400
C	2.23096000	-1.25750800	-0.00038900
C	2.95485300	1.36244100	0.00058900
H	0.81086100	1.66008100	0.00244800
C	3.58581800	-0.94354600	-0.00129200
H	1.90605200	-2.29140700	-0.00077000
C	3.95455000	0.39683500	-0.00070200
H	3.19415200	2.41681100	0.00109500
H	4.32699500	-1.73062500	-0.00228300
H	4.99818300	0.68183500	-0.00141400

Cartesian coordinates of the optimized geometry for **9b** at B3LYP-D3BJ/TZVP,aug-cc-pVTZ-PP level of theory (number of imaginary frequencies = 0):

Se	-1.31713100	-0.67062400	0.00010400
C	-3.55576700	0.53332600	-0.00010700
H	-4.61827400	0.74509000	-0.00015200
N	-1.29411900	1.15770800	-0.00002200
C	-2.54539400	1.54848700	-0.00011300
H	-2.76086900	2.61002600	-0.00017400
N	-3.14163400	-0.70927500	-0.00002200
N	1.47221500	-0.18856000	-0.00016400
C	2.37598800	-1.17305600	-0.00022100
C	1.91830400	1.07260300	-0.00007100
C	3.74793900	-0.94913600	0.00005300
H	1.98072500	-2.18230000	-0.00023900
C	3.27122000	1.39499700	0.00025100
H	1.14852800	1.83322200	0.00003100
C	4.20383600	0.36431600	-0.00007600
H	4.43552800	-1.78326500	-0.00022900
H	3.58080100	2.43076900	0.00013900
H	5.26402800	0.57933300	0.00023100

Cartesian coordinates of the optimized geometry for **9c** at B3LYP-D3BJ/TZVP,aug-cc-pVTZ-PP level of theory (number of imaginary frequencies = 0):

Te	-1.05540000	-0.62857300	-0.00017000

C	-3.37140000	0.80698600	-0.00009300
H	-4.41230500	1.12404600	-0.00021800
N	-1.05547800	1.40437800	0.00076800
C	-2.29975400	1.78573800	0.00067300
H	-2.51552100	2.85157500	0.00104300
N	-3.08126300	-0.46003500	-0.00056000
N	1.58704600	-0.06621500	0.00064400
C	2.44441700	-1.09172900	0.00088700
C	2.07569100	1.18011400	-0.00012800
C	3.82264300	-0.92139400	0.00023100
H	2.00361500	-2.08221400	0.00143000
C	3.44080200	1.44223700	-0.00081000
H	1.33160900	1.96514100	-0.00019200
C	4.32988400	0.37353800	-0.00036100
H	4.47647000	-1.78197200	0.00068400
H	3.79330400	2.46399000	-0.00129000
H	5.39778200	0.54540600	-0.00096900

Cartesian coordinates of the optimized geometry for **10a** at B3LYP-D3BJ/TZVP,aug-cc-pVTZ-PP level of theory (number of imaginary frequencies = 0):

C	-2.69073100	-1.67003400	0.00425600
C	-1.38501000	-1.28226400	0.00312100
H	-3.00709200	-2.70129500	0.00694400
H	-0.49800400	-1.88971000	0.00460300
N	-3.02413800	0.61340500	-0.00153400
C	-3.60353200	-0.59559700	0.00161400
H	-4.68017600	-0.62742700	0.00179800
O	-3.57067300	1.74318100	-0.00436600
S	-1.22900000	0.44426400	-0.00131800
N	1.54292400	-0.13674000	0.00016600
C	2.22992200	-0.05868100	-1.14467300
C	2.22926800	-0.05059900	1.14482000
C	3.61008700	0.10005700	-1.19615300
H	1.64492500	-0.12313400	-2.05412700
C	3.60940300	0.10850400	1.19596700
H	1.64374700	-0.10863700	2.05437000
C	4.31354800	0.18496900	-0.00017700
H	4.11506500	0.15800400	-2.15009700
H	4.11383600	0.17318400	2.14976600
H	5.38786100	0.31055400	-0.00031200

Cartesian coordinates of the optimized geometry for **10b** at B3LYP-D3BJ/TZVP,aug-cc-pVTZ-PP level of theory (number of imaginary frequencies = 0):

C	2.33141600	-1.85891200	-0.00230300
C	1.05265300	-1.39900100	-0.00192000
H	2.56971000	-2.91276100	-0.00364400
H	0.14378700	-1.97468700	-0.00267600
N	2.94784700	0.39036400	0.00057100
C	3.35491300	-0.88284200	-0.00098200
H	4.41915400	-1.05856500	-0.00109700
O	3.65896100	1.42413900	0.00186200
Se	0.98651700	0.47141000	0.00050900
N	-1.71313300	0.05619500	0.00005100
C	-2.40001100	0.03494500	1.14727000
C	-2.40015500	0.03924600	-1.14715700
C	-3.78830200	-0.00647900	1.19685500
H	-1.80866900	0.05540900	2.05438500
C	-3.78844900	-0.00198400	-1.19673100
H	-1.80892400	0.06309300	-2.05426200
C	-4.49547300	-0.02858700	0.00005900
H	-4.29701300	-0.02307400	2.15030500
H	-4.29727500	-0.01500200	-2.15017500

H	-5.57659100	-0.05971600	0.00006700

Cartesian coordinates of the optimized geometry for **10c** at B3LYP-D3BJ/TZVP,aug-cc-pVTZ-PP level of theory (number of imaginary frequencies = 0):

C	2.10048600	-2.03879900	-0.00042200
C	0.82942900	-1.56079900	-0.00048100
H	2.30162000	-3.10297700	-0.00063700
H	-0.07230900	-2.15119700	-0.00077300
N	2.92160900	0.17196900	0.00022400
C	3.19558100	-1.13317100	-0.00004700
H	4.23815900	-1.41782600	0.00000300
O	3.76893800	1.11061700	0.00054600
Te	0.79800000	0.50821400	-0.00004600
N	-1.82907600	0.12138900	-0.00030500
C	-2.50621600	0.04583900	1.15157700
C	-2.50693400	0.04655300	-1.15180300
C	-3.88568200	-0.10933400	1.19806800
H	-1.91379400	0.11252300	2.05530700
C	-3.88643000	-0.10857800	-1.19752700
H	-1.91507600	0.11378300	-2.05586100
C	-4.58781000	-0.18843600	0.00046500
H	-4.39221500	-0.16625900	2.15096300
H	-4.39356300	-0.16489600	-2.15013900
H	-5.66260600	-0.30835500	0.00075900

Cartesian coordinates of the optimized geometry for **11a** at B3LYP-D3BJ/TZVP,aug-cc-pVTZ-PP level of theory (number of imaginary frequencies = 0):

N	2.13235400	-1.25781100	0.46633100
C	1.70280200	-1.02240500	-0.73816500
C	2.32957200	-0.07722400	1.14385700
C	2.04459200	1.06138700	0.45317400
H	1.47347900	-1.79046100	-1.45995200
H	2.68929300	-0.10803600	2.16045900
H	2.10248600	2.08248000	0.78725300
S	1.49904200	0.66413700	-1.14022000
C	-1.14755200	-0.25562300	1.14249000
C	-2.18128700	1.33298200	0.03010600
C	-2.56910300	0.28393500	-0.74831600
H	-0.52671800	-0.73950300	1.87934100
H	-2.46160000	2.36142500	-0.13598000
H	-3.19493300	0.29232200	-1.62394600
S	-1.90210000	-1.18861000	-0.12681900
N	-1.37624300	1.02281700	1.10032300

Cartesian coordinates of the optimized geometry for **11b** at B3LYP-D3BJ/TZVP,aug-cc-pVTZ-PP level of theory (number of imaginary frequencies = 0):

N	2.15680000	1.53181900	1.10006000
C	1.98453100	0.27195800	1.33324200
C	2.39906100	1.80036700	-0.22842700
C	2.42024000	0.75268600	-1.09662400
H	1.78046600	-0.13800600	2.31070300
H	2.55814000	2.82770900	-0.52030300
H	2.59479900	0.78378000	-2.15878600
Se	2.11063200	-0.84896200	-0.18917400
C	-1.32821800	1.00665700	-0.18192700
C	-3.54998800	1.10421400	-0.13908400
C	-3.50093100	-0.24182500	0.04922200
H	-0.30639000	1.34485100	-0.24965700
H	-4.46708900	1.67160000	-0.19539000
H	-4.33151900	-0.91754000	0.16419200

Se	-1.72724700	-0.82574300	0.08276100
N	-2.35846700	1.78292600	-0.26450800

Cartesian coordinates of the optimized geometry for **11c** at B3LYP-D3BJ/TZVP,aug-cc-pVTZ-PP level of theory (number of imaginary frequencies = 0):

N	1.85185700	1.71525200	1.37303400
C	1.74417200	0.43822200	1.50248500
C	2.22317000	2.16662400	0.12560100
C	2.45103200	1.28754000	-0.88767200
H	1.45234000	-0.03941700	2.42716400
H	2.31964200	3.23683600	0.00168600
H	2.74363800	1.55094000	-1.89164100
Te	2.17302800	-0.64920300	-0.24554300
C	-1.41911000	1.24998700	-0.44569400
C	-3.67567300	1.31092900	-0.29698000
C	-3.68056500	0.00147000	0.06944000
H	-0.42579000	1.63663400	-0.62381700
H	-4.58104700	1.89512500	-0.39317100
H	-4.56121800	-0.57795700	0.29727200
Te	-1.75671400	-0.73827000	0.13271500
N	-2.48815500	1.95872100	-0.56639600

Cartesian coordinates of the optimized geometry for **12a** at B3LYP-D3BJ/TZVP,aug-cc-pVTZ-PP level of theory (number of imaginary frequencies = 0):

N	3.09994700	-0.90843300	1.00089600
C	1.91849200	-0.79295200	0.47209500
C	3.97026400	-0.00235000	0.44099500
C	3.44629300	0.80961400	-0.51977600
H	1.05463900	-1.38119200	0.73345600
H	4.99408800	0.02077600	0.78070200
H	3.94058200	1.58053300	-1.08567300
S	1.77000500	0.43930900	-0.76002000
N	-1.27341000	-0.57021100	-0.10446700
C	-1.65656900	0.50936800	0.51274200
C	-3.56506500	-0.63734300	-0.44965200
C	-2.35222200	-1.22355800	-0.65139300
H	-0.98463700	1.18743100	1.01552300
H	-4.53437400	-0.96671900	-0.78168200
H	-2.19047700	-2.13956400	-1.19738500
S	-3.37205200	0.81522600	0.47439400

Cartesian coordinates of the optimized geometry for **12b** at B3LYP-D3BJ/TZVP,aug-cc-pVTZ-PP level of theory (number of imaginary frequencies = 0):

N	3.28250800	1.45240100	0.86036100
C	2.13364400	0.86372300	0.78059200
C	4.25252600	0.82818300	0.10696800
C	3.89523200	-0.28093000	-0.59541900
H	1.23894200	1.18251200	1.29055900
H	5.24729000	1.24887800	0.11018400
H	4.52866300	-0.88058300	-1.22753800
Se	2.08647000	-0.66856100	-0.32654800
N	-0.91922600	-0.19776800	0.51102600
C	-1.50365900	0.44676800	-0.44927800
C	-3.13465700	-0.70108000	1.08120300
C	-1.80800900	-0.82802400	1.35172200
H	-0.97142600	0.99923100	-1.20905200
H	-3.95986400	-1.12313300	1.62921800
H	-1.41167000	-1.39216600	2.18257500
Se	-3.38721600	0.35123700	-0.43905100

Cartesian coordinates of the optimized geometry for **12c** at B3LYP-D3BJ/TZVP,aug-cc-pVTZ-PP level of theory (number of imaginary frequencies = 0):

N	3.30412100	1.94869100	0.56510000
C	2.18533900	1.31043500	0.50799200
C	4.41602800	1.24257600	0.14893600
C	4.29299100	-0.04072000	-0.28411500
H	1.23859000	1.73917400	0.80465900
H	5.36310500	1.76424300	0.18999100
H	5.11373000	-0.65331900	-0.62400300
Te	2.31931800	-0.65185900	-0.20470100
C	-1.38418200	0.23797000	-0.66107500
C	-2.69859100	-0.62882600	1.44854300
C	-1.34325100	-0.72387400	1.39221600
N	-0.66195000	-0.26774000	0.28551000
H	-0.95490200	0.62525300	-1.57482500
H	-3.31620600	-0.95591300	2.26959000
H	-0.74937700	-1.14830500	2.19039100
Te	-3.43470600	0.23795200	-0.26693500

Cartesian coordinates of the optimized geometry for **13a** at B3LYP-D3BJ/TZVP,aug-cc-pVTZ-PP level of theory (number of imaginary frequencies = 0):

N	-1.81447600	-0.82681100	0.53057200
C	-1.60331700	0.48900200	0.42096600
C	-2.94420700	-1.26055200	-0.17764200
C	-3.58039900	-0.26355600	-0.82508100
H	-0.76108300	0.99359800	0.88782600
H	-3.17943700	-2.30903200	-0.12892000
H	-4.46738000	-0.33374900	-1.42967200
O	-1.07774500	-1.65197500	1.20002500
S	-2.78348100	1.25846700	-0.57195900
C	1.60300900	-0.48895000	0.42036400
C	3.58078700	0.26356300	-0.82462000
C	2.94437700	1.26056700	-0.17741200
N	1.81439900	0.82678600	0.53038400
H	0.76079300	-0.99363900	0.88712400
H	4.46784300	0.33376600	-1.42909800
H	3.17974100	2.30900500	-0.12840700
S	2.78351200	-1.25846400	-0.57195200
O	1.07750300	1.65194200	1.19967200

Cartesian coordinates of the optimized geometry for **13b** at B3LYP-D3BJ/TZVP,aug-cc-pVTZ-PP level of theory (number of imaginary frequencies = 0):

N	1.64778300	1.13392500	0.71598400
C	1.66351500	-0.19638000	0.62110100
C	2.66558100	1.79015900	-0.00087600
C	3.50257600	0.98722300	-0.68088500
H	0.91983800	-0.82285500	1.10638000
H	2.67179300	2.86436000	0.07185900
H	4.34123000	1.29231300	-1.28224900
O	0.78251800	1.81748000	1.39370300
Se	3.04247700	-0.81571100	-0.46156400
C	-1.66351500	0.19630100	0.62115600
C	-3.50257400	-0.98716100	-0.68099200
C	-2.66564400	-1.79016200	-0.00097600
N	-1.64783100	-1.13400300	0.71595000
H	-0.91981900	0.82274100	1.10646300
H	-4.34123300	-1.29221800	-1.28236500
H	-2.67186100	-2.86436800	0.07168300
Se	-3.04243000	0.81576900	-0.46153900
O	-0.78262300	-1.81764000	1.39367400

Cartesian coordinates of the optimized geometry for **13c** at B3LYP-D3BJ/TZVP,aug-cc-pVTZ-PP level of theory (number of imaginary frequencies = 0):

N	3.09673500	1.59352300	0.52969900
C	2.18977400	0.69592500	0.92184500
C	3.82054500	1.28196100	-0.65059800
C	3.55734400	0.11863200	-1.26624700
H	1.65283800	0.85798000	1.84049300
H	4.53296600	2.04122400	-0.93328300
H	4.05184000	-0.20353200	-2.16926300
O	3.34229200	2.69498000	1.14261800
Te	2.06586300	-0.96146300	-0.28737100
C	-1.33001600	0.37580100	0.45871000
C	-3.39290900	-1.26284500	0.15197700
C	-2.27171100	-1.75304000	0.70571900
N	-1.17541600	-0.88147600	0.86945600
H	-0.51093400	1.06817500	0.55950100
H	-4.29243800	-1.82910600	-0.02377700
H	-2.09474700	-2.75787100	1.05454000
Te	-3.18739200	0.73627000	-0.33463600
O	-0.08072000	-1.34370500	1.39408300

Cartesian coordinates of the optimized geometry for **14a** at B3LYP-D3BJ/TZVP,aug-cc-pVTZ-PP level of theory (number of imaginary frequencies = 0):

S	-1.68828700	0.95436300	0.00589300
C	-3.65483600	-0.44009300	-0.01394700
C	-2.52959300	-1.30453800	-0.00087600
H	-4.68999800	-0.74754700	-0.02508100
H	-2.56017300	-2.38404100	-0.00011100
N	-1.36348700	-0.68571300	0.01126300
N	-3.35028200	0.84443500	-0.01186800
S	1.69832700	-0.96189600	0.00856900
C	2.51427900	1.30632600	0.00078600
C	3.64900900	0.45437400	-0.01666900
H	2.53257700	2.38610200	0.00245800
H	4.68068500	0.77316000	-0.03107900
N	1.35534600	0.67427000	0.01610200
N	3.35886800	-0.83350200	-0.01454400

Cartesian coordinates of the optimized geometry for **14b** at B3LYP-D3BJ/TZVP,aug-cc-pVTZ-PP level of theory (number of imaginary frequencies = 0):

Se	-1.73252600	0.87510500	-0.00000700
C	-3.51625200	-0.93125700	0.00003000
C	-2.25662100	-1.61025500	-0.00002300
H	-4.47293700	-1.43860000	0.00004300
H	-2.15110100	-2.68787300	-0.00003900
N	-1.17741700	-0.86541200	-0.00003300
N	-3.48093800	0.37961800	0.00002400
Se	1.73253200	-0.87510500	0.00000900
C	2.25660500	1.61024400	-0.00000500
C	3.51624400	0.93126100	-0.00000200
H	2.15110000	2.68786400	0.00004100
H	4.47291700	1.43862700	-0.00000500
N	1.17741000	0.86539900	0.00000600
N	3.48093800	-0.37960400	-0.00000900

Cartesian coordinates of the optimized geometry for **14c** at B3LYP-D3BJ/TZVP,aug-cc-pVTZ-PP level of theory (number of imaginary frequencies = 0):

Te	-1.73144500	0.82777800	0.00024000
C	-3.20677500	-1.48353200	-0.00110800
C	-1.82421800	-1.91998400	-0.00029500
H	-4.01560100	-2.21041200	-0.00165800
H	-1.55735200	-2.97302800	-0.00013700
N	-0.88172400	-1.02417800	0.00041100
N	-3.47530700	-0.21176200	-0.00076200
Te	1.73168000	-0.82779800	0.00026400
C	1.82374800	1.91987400	-0.00018900
C	3.20614700	1.48396300	-0.00095800
H	1.55631800	2.97272400	-0.00024900
H	4.01502100	2.21074800	-0.00170600
N	0.88125800	1.02372900	0.00030500
N	3.47520200	0.21208300	-0.00097600

Cartesian coordinates of the optimized geometry for **15a** at B3LYP-D3BJ/TZVP,aug-cc-pVTZ-PP level of theory (number of imaginary frequencies = 0):

S	-2.09949200	0.37111100	-0.85218100
C	-1.31606900	0.83973400	1.54274100
C	-1.76450900	1.48231100	0.42884000
C	-1.22020000	-0.55751800	1.39054000
H	-1.04612300	1.34526600	2.45598600
H	-1.89949500	2.53982200	0.27856500
H	-0.88206900	-1.28850900	2.10494500
N	-1.59167300	-1.01375200	0.18708000
O	-1.57765200	-2.18659800	-0.25004200
S	1.37797200	-0.59509400	-1.18079600
C	2.02658300	0.80812900	0.88915600
C	2.33408900	-0.53001600	1.19750700
C	2.03638100	-1.40520700	0.19481400
H	2.15475800	1.68516300	1.50065800
H	2.75793100	-0.82714000	2.14363300
H	2.16622600	-2.47435600	0.18131900
N	1.50565000	0.99229500	-0.33351800
O	1.11735200	2.05273300	-0.87170700

Cartesian coordinates of the optimized geometry for **15b** at B3LYP-D3BJ/TZVP,aug-cc-pVTZ-PP level of theory (number of imaginary frequencies = 0):

Se	-1.94000600	-0.98258700	-0.06346500
C	-3.77290700	0.94046500	-0.53446600
C	-3.59320200	-0.39615200	-0.71019300
C	-2.70355100	1.62485100	0.07970600
H	-4.66499900	1.46474300	-0.84346300
H	-4.28838800	-1.08477800	-1.16325900
H	-2.65118500	2.67836400	0.30326000
N	-1.63955800	0.89337900	0.42277600
O	-0.59284300	1.30056500	0.98422000
Se	1.94120000	0.98297200	-0.06325700
C	2.70136600	-1.62553900	0.08021300
C	3.77094900	-0.94281600	-0.53540000
C	3.59301200	0.39404200	-0.71111300
H	2.64812900	-2.67889200	0.30436900
H	4.66183500	-1.46853600	-0.84541700
H	4.28851800	1.08143600	-1.16556500
N	1.63872300	-0.89266800	0.42435600
O	0.59251000	-1.29800400	0.98780500

Cartesian coordinates of the optimized geometry for **15c** at B3LYP-D3BJ/TZVP,aug-cc-pVTZ-PP level of theory (number of imaginary frequencies = 0):

Te	-1.36461700	0.99162100	-0.11198100

C	-3.83444700	-0.46702700	0.41056600
C	-3.39883300	0.81196800	0.30771800
C	-2.88042000	-1.50663300	0.23416500
H	-4.86362600	-0.72417400	0.62266500
H	-4.02391100	1.68575800	0.42159800
H	-3.09676700	-2.56338900	0.29732400
N	-1.63821600	-1.15500300	-0.02147800
O	-0.66917900	-2.00453500	-0.20053600
Te	1.31744600	-0.75255000	-0.49917000
C	2.95675500	0.98908700	1.18370400
C	2.32103100	0.06175300	2.06167900
C	1.47829800	-0.87521900	1.56672300
H	3.62201800	1.78163500	1.49661000
H	2.52067600	0.13027700	3.12406400
H	0.94321500	-1.62679300	2.12389900
N	2.71269800	0.86952400	-0.11768000
O	3.16613000	1.60450200	-1.03940800

4. Supporting Information References

[1] M. J. Frisch, G. W. Trucks, H. B. Schlegel, G. E. Scuseria, M. A. Robb, J. R. Cheeseman, G. Scalmani, V. Barone, G. A. Petersson, H. Nakatsuji, X. Li, M. Caricato, A. V. Marenich, J. Bloino, B. G. Janesko, R. Gomperts, B. Mennucci, H. P. Hratchian, J. V. Ortiz, A. F. Izmaylov, J. L. Sonnenberg, D. Williams-Young, F. Ding, F. Lipparini, F. Egidi, J. Goings, B. Peng, A. Petrone, T. Henderson, D. Ranasinghe, V. G. Zakrzewski, J. Gao, N. Rega, G. Zheng, W. Liang, M. Hada, M. Ehara, K. Toyota, R. Fukuda, J. Hasegawa, M. Ishida, T. Nakajima, Y. Honda, O. Kitao, H. Nakai, T. Vreven, K. Throssell, J. A. Montgomery, Jr.; , J. E. Peralta, F. Ogliaro, M. J. Bearpark, J. J. Heyd, E. N. Brothers, K. N. Kudin, V. N. Staroverov, T. A. Keith, R. Kobayashi, J. Normand, K. Raghavachari, A. P. Rendell, J. C. Burant, S. S. Iyengar, J. Tomasi, M. Cossi, J. M. Millam, M. Klene, C. Adamo, R. Cammi, J. W. Ochterski, R. L. Martin, K. Morokuma, O. Farkas, J. B. Foresman, D. J. Fox, *Gaussian 16, Revision A.03*, Gaussian, Inc., Wallingford CT, **2016**.

[2] H.-J. Werner, P. J. Knowles, G. Knizia, F. R. Manby, M. Schütz, a. others, *MOLPRO, version 2012.1, a package of ab initio programs*, see http://www.molpro.net, **2012**.

[3] S. Grimme, *J. Chem. Phys.* **2006**, *124*, 034108.

[4] S. Grimme, S. Ehrlich, L. Goerigk, *J. Comp. Chem.* **2011**, *32*, 1456-1465.

[5] B. Miehlich, A. Savin, H. Stoll, H. Preuss, *Chem. Phys. Lett.* **1989**, *157*, 200-206.

[6] A. D. Becke, *Phys. Rev. A* **1988**, *38*, 3098-3100.

[7] C. Lee, W. Yang, R. G. Parr, *Phys. Rev. B* **1988**, *37*, 785-789.

[8] G. D. Purvis III, R. J. Bartlett, *J. Chem. Phys.* **1982**, *76*, 1910-1918.

[9] G. Jansen, *WIREs Comput. Mol. Sci.* **2014**, *4*, 127-144.

[10] A. Hesselmann, G. Jansen, M. Schütz, *J. Chem. Phys.* **2005**, *122*, 014103.

[11] K. Szalewicz, K. Patkowski, B. Jeziorski, *Struct. Bond* **2005**, *116*, 43-117.

[12] B. Jeziorski, R. Moszynski, K. Szalewicz, *Chem. Rev.* **1994**, *94*, 1887-1930.

[13] J. P. Perdew, K. Burke, M. Ernzerhof, *Phys. Rev. Lett.* **1997**, *78*, 1396.

[14] J. P. Perdew, K. Burke, M. Ernzerhof, *Phys. Rev. Lett.* **1996**, *77*, 3865.
